# Molecular regulation of PGC-1α: from protein-protein interactions and post-translational modifications to pharmacological modulation

**DOI:** 10.1007/s00109-026-02694-6

**Published:** 2026-06-19

**Authors:** William Q. Rios, Carlos M. Silva, Rita Ferreira, José R. B. Gomes

**Affiliations:** 1 Department of Chemistry, CICECO—Aveiro Institute of Materials, University of Aveiro, Aveiro, 3810-193 Portugal; 2 Department of Chemical Engineering, Faculty of Sciences and Technology, CERES, University of Coimbra, Rua Sílvio Lima, Polo II, Coimbra, 3030-790 Portugal; 3 Department of Chemistry, LAQV–REQUIMTE, University of Aveiro, Aveiro, 3810-193 Portugal

**Keywords:** Transcriptional coactivator, Energy metabolism, Post-translational control, Intrinsically disordered protein

## Abstract

Peroxisome proliferator-activated receptor gamma coactivator 1α (PGC-1α) is a master transcriptional coactivator responsible for regulating cellular energy metabolism and mitochondrial biogenesis across high-energy tissues such as the heart, skeletal muscle, and brown adipose tissue. To orchestrate its regulatory functions, PGC-1α interacts with a diverse array of transcription factors such as peroxisome proliferator-activated receptors (PPARs), estrogen-related receptors (ERRs), and nuclear respiratory factors (NRFs), which is facilitated by its dynamic three-dimensional structure, the presence of distinct functional domains, and the ability to be modulated via post-translational modifications. This review examines the protein’s interactions with key nuclear receptors and the biological consequences of these complexes, including the regulation of thermogenesis, gluconeogenesis, and fatty acid oxidation. Furthermore, we discuss the extensive post-translational modifications—including phosphorylation, acetylation, methylation, O-GlcNAcylation, and ubiquitination—that tightly regulate PGC-1α stability and coactivation efficiency. Finally, this review highlights recent progress in the identification of small molecule modulators, such as the activator ZLN005 and the inhibitor SR18292, evaluating their physiological outcomes and potential as therapeutic agents for metabolic disorders and cancer, while addressing the challenges posed by the protein’s structural disorder in drug discovery.

## Introduction

 Peroxisome proliferator-activated receptor gamma (PPARγ) coactivator 1 (PGC-1), consists of a family of transcriptional coactivator proteins responsible for regulating the cellular energy metabolism [1]. This family includes members PGC-1α, PGC-1β [2–4], and PGC-1-related coactivator (PRC) [5]. As coactivators, these proteins do not bind to DNA directly but instead interact with transcription factors or nuclear receptors, forming complexes that enhance the rate of transcription of target genes by recruiting additional regulatory factors to specific DNA promoter regions [6, 7].

The main member of the PGC-1 family, PGC-1α, is encoded by the PPARGC1A gene and was first cloned and characterized by Puigserver et al. [6] in 1998. Initially recognized for its role in coactivating PPARγ to induce thermogenic gene expression in brown adipocytes, numerous other tissue-specific roles have been attributed to PGC-1α over the years [7]. Moreover, several PGC-1α isoforms obtained by alternative promoter transcription initiation and/or alternative splicing events have been identified [1], such as N-terminal truncated variants [8, 9], and brain-specific variants [10, 11]. The originally characterized isoform of the protein was later designated PGC-1α1, though it is commonly referred to as PGC-1α for simplicity.

In general, PGC-1α is highly expressed in energy-demanding tissues such as brown adipose tissue (BAT), skeletal muscle, heart, liver, and brain [12, 13]. A high-level overview of the biological activities of PGC-1α in these tissues is given in the following paragraphs.

In BAT, PGC-1α promotes mitochondrial biogenesis and the expression of uncoupling protein 1 (UCP1), an inner mitochondrial membrane protein largely responsible for the heat-generating properties of this tissue [14]. UCP1 uncouples the respiratory chain, allowing protons to pass from the intermembrane space to the mitochondrial matrix without ATP synthesis, thereby dissipating the proton gradient to generate heat [15]. The expression of PGC-1α in BAT is upregulated in response to external stimuli such as cold-exposure and β_3_-adrenergic receptor activation, which drive the thermogenic adaptation [6].

In skeletal muscle, PGC-1α is preferentially expressed in type I (slow-twitch) muscle fibers, which have a high mitochondrial content and rely primarily on oxidative phosphorylation for ATP production, in contrast with type II (fast-twitch) fibers, which generate ATP mainly via glycolysis [16]. Consistent with this fiber-type specificity, overexpression of PGC-1α in type II fibers induces their transcriptional reprogramming toward a fatigue-resistant, oxidative type I fibers, characterized by upregulation of mitochondrial oxidative metabolism genes and slow-twitch myofibrillar protein isoforms [16]. In addition to promoting oxidative remodeling, overexpression of PGC-1α or its homolog PGC-1β inhibits muscle protein degradation, increasing total protein content in myotubes, by slowing the rate of both lysosomal and proteasomal proteolysis [17]. In the latter case, the effect is at least in part mediated by a suppression of muscle-specific ubiquitin ligases associated with muscle atrophy [17].

PGC-1α transcription in skeletal muscle is strongly induced by physical activity, particularly endurance exercise [18], which elicits a rapid but transient increase in PGC-1α mRNA and protein levels that is thought to drive late-phase adaptations to aerobic training, such as enhanced oxidative capacity via mitochondrial biogenesis [19]. While the canonical PGC-1α isoform is primarily associated with endurance exercise, truncated isoforms have been reported to be induced by both aerobic and resistance exercise modalities [20, 21]. Among these, PGC-1α4 has been proposed to promote skeletal muscle hypertrophy through downregulation of myostatin—a potent inhibitor of muscle growth—an effect supported by transgenic animal models overexpressing skeletal muscle PGC-1α4 [22]. However, whether truncated isoforms contribute to hypertrophic adaptations in human skeletal muscle remains unresolved due to conflicting results in studies with human subjects [23], which is further complicated by differences in exercise protocols and experimental design.

PGC-1α expression in the heart naturally rises at the time of birth, triggering the metabolic transition of the mammalian heart from a fetal glycolysis-dominant state to the postnatal phenotype, where mitochondrial fatty acid oxidation becomes the main pathway for energy production [24, 25]. Consistent with this effect, in cultured cardiac myocytes, forced expression of PGC-1α induces the expression of genes encoding components of the oxidative phosphorylation complex and enhances mitochondrial biogenesis, leading to increased mitochondrial coupled respiration [26].

In the liver, PGC-1α is induced by fasting and other insulin-deficient states, where it promotes hepatic gluconeogenesis by increasing the expression of key enzymes of the gluconeogenesis pathway [27]. These effects are counteracted by insulin, which suppresses hepatic gluconeogenesis in part by reducing the ability of PGC-1α to coactivate transcription factors that bind to promoter regions of gluconeogenic genes [28].

In the brain, PGC-1α is a critical component of the oxidative stress defense by regulating the expression of enzymes responsible for detoxifying reactive oxygen species (ROS) [29]. ROS, such as superoxide ($\:{\mathrm{O}}_{2}^{-}$), are natural byproducts of mitochondrial metabolism capable of damaging chain reactions that continue until the radical electron is neutralized [30]. PGC-1α expression is induced in response to oxidative stressors, leading to an increased production of ROS-detoxifying enzymes that protect cells from oxidative damage [29]. Additionally, PGC-1α contributes to neuronal function by promoting mitochondrial biogenesis and increasing the energy production capacity of neurons, necessary for dendritic spine formation and maintenance of synapses [31].

Thus, PGC-1α plays a central role in regulating energy metabolism across multiple tissues and has been extensively studied in various biological contexts. While numerous reviews have been published in recent years, most have focused on its involvement in pathological processes, including neurodegenerative disorders [32–38], cancer [39–42], heart failure [43, 44], kidney disorders [45–47] and type 2 diabetes [48], whereas others have emphasized mitochondrial lifecycle regulation [49–52] or tissue-specific functions [53–56]. More recently, Qian et al. [57] provided a comprehensive overview of the broader PGC-1 family, highlighting the involvement of PGCs in various signaling networks under both physiological and pathological conditions, and Cao et al. [58] reviewed the roles of PGC-1α in cellular differentiation across several tissues, such as bone and epithelial tissues. In contrast to these publications, the present review focuses specifically on PGC-1α at the molecular level, emphasizing its structural features, regulatory protein-protein interactions, post-translational modifications, and modulation by synthetic and natural small molecules, with the goal of supporting future therapeutic development. Accordingly, we discuss the functional domains of PGC-1α, its interactions with various protein partners and their biological consequences, the post-translational modifications regulating its activity, and the known synthetic or natural small molecule modulators that enhance or inhibit PGC-1α function.

## Functional domains of PGC-1α and structure

**Fig. 1:**
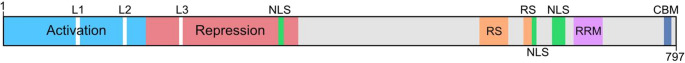
Functional domains of full-length murine PGC-1α

PGC-1α exhibits bi-functional activity, participating in both transcription initiation and post-transcriptional processes. The full-length human protein (UniProt Q9UBK2 [59]) consists of 798 amino acids, while the murine version (UniProt O70343 [60]), which has 95% homology with the human PGC-1α [61], contains 797 amino acids. Figure 1 summarizes the key functional domains of the protein. The positions shown correspond to the murine version, as most experiments have been performed with it, and this ensures consistency with the descriptions that follow. Due to the high degree of homology between human and murine PGC-1α, the corresponding positions in the human protein can be inferred by offsetting them by 1–2 amino acids.

### N-terminal region

The N-terminal region of murine PGC-1α (amino acids 1–170) harbors a transcriptional activation domain that binds to other transcriptional coactivators such as steroid receptor coactivator-1 (SRC-1) and CREB-binding protein (CPB)/p300 [62]. Since PGC-1α itself has little to no histone acetyltransferase (HAT) activity, its coactivation function depends largely on recruitment of these HAT-containing proteins [62]. Histone acetylation reduces the positive charges on lysine residues of histones, which decreases their affinity to negatively charged DNA, and loosens the chromatin structure, making it more accessible to the transcriptional machinery [63]. It is hypothesized that unbound PGC-1α has low transcriptional activity, and docking to a transcription factor induces conformational changes that facilitates the assembly of HAT-containing coactivators to initiate target gene transcription [62].

The N-terminal region also contains three leucine-rich sequences: L1 (LLAVL, amino acids 86–90), L2 (LKKLL, amino acids 142–146), and L3 (LLKYL, amino acids 209–213) [64]. The L2 motif conforms to the LXXLL consensus sequence (where X represents any amino acid) and mediates interactions with ligand-activated nuclear receptors such as the estrogen receptor α (ERα) and PPARα [7]. The L3 motif, which corresponds to an inverted LXXLL, serves as the primary binding site for the estrogen-related receptor α (ERRα) [65]. Additionally, it has a secondary role in the interaction with the glucocorticoid receptor (GR) [64], acting as a complementary binding site alongside L2.

In addition to participating in chromatin remodeling events by recruiting coactivators with HAT activity, PGC-1α also interacts with subunits of the SWI/SNF (switching defective/sucrose non-fermenting) family of ATP-dependent chromatin remodelers [66]. SWI/SNF complexes are multiprotein assemblies that recognize histone modifications and use ATP hydrolysis to reposition or evict nucleosomes, thereby increasing chromatin accessibility at target loci for DNA-binding transcription factors [67]. Within these complexes, BAF60 (BRG1/BRM-associated factor 60) subunits function as adaptors that connect the remodeling core to transcriptional regulators [68]. Genome-wide and biochemical analyses have shown that the BAF60a isoform—whose tissue distribution overlaps with PGC‑1α—binds broadly to the central portion of PGC‑1α (approximately amino acids 180–560), whereas the N-terminal activation domain is not required for this interaction [66]. Functionally, BAF60a promotes the expression of fatty acid oxidation genes in the liver, an effect that is attenuated in PGC‑1α-null hepatocytes, indicating that PGC‑1α is required for efficient BAF60a–mediated transcriptional activation of these pathways [66]. In parallel, BAF60 isoforms associate with several nuclear receptors—including PPARα [66], PPARγ [69], and GR [70]—in a ligand-independent manner, suggesting that cooperative interactions between nuclear receptors, PGC‑1α, and SWI/SNF complexes contribute to coordinated chromatin remodeling at metabolic gene loci.

### Central region

The central region of PGC-1α (amino acids 170–350) contains a negative regulatory domain, also called repression domain. Its repressive role is supported by findings that mutated versions of the protein lacking this segment exhibit higher transcriptional coactivation activity than the full-length protein [62]. The central region also contains a nuclear localization signal (NLS) at amino acids 326–333, with two additional NLS sequences located in the C-terminal region (amino acids 627–633 and 651–667) [7]. NLS motifs are rich in basic amino acids, typically arginine and lysine, and are recognized by nuclear trafficking proteins of the importin superfamily, which mediate the transport of proteins from the cytosol to the nucleus through the nuclear pore complex [71]. These sequences appear to be important for the primarily nuclear localization of full-length PGC-1α, as evidenced by the fact that a truncated alternative splicing variant (NT-PGC-1α), containing only the first 267 amino acids of the full-length protein and three additional splice-derived residues, is found predominantly in the cytosol under basal conditions [8].

In addition to changes in cellular localization, the absence of part of the central region and the C-terminal domain (CTD) in NT‑PGC‑1α has broader functional consequences, as it restricts a subset of protein–protein interactions available to the full-length protein [72, 73]. This difference is exemplified by the transcription factor Twist‑1, which acts as a selective negative regulator of PGC‑1α activity in brown adipose tissue. Twist‑1 binds to a region within amino acids 353–797 of PGC‑1α—absent from NT‑PGC‑1α—and suppresses induction of genes involved in fatty acid oxidation and mitochondrial uncoupling, while leaving other PGC‑1α target genes largely unaffected [73]. This isoform‑specific repression illustrates how the presence of certain domains shapes regulatory output, distinguishing the functional repertoires of full‑length PGC‑1α and NT‑PGC‑1α despite their extensive sequence overlap and shared ability to activate overlapping subsets of target genes.

### C-terminal region

The C-terminal region of PGC-1α contains two arginine/serine-rich (RS) domains located between amino acids 565–598 and 617–631, and an RNA recognition motif (RRM), which confers low-specificity RNA-binding ability, spanning amino acids 677–710 [74, 75]. RS domains are characteristic of SR (serine/arginine-rich) proteins, which participate in pre-mRNA processing (splicing) and mRNA export. These proteins typically contain one or two RRMs in their N-terminal region [76], and the RS domain can also interact with pre-mRNAs within the cellular splicing machinery [77].

Compared to its transcriptional activation functions, the contribution of PGC‑1α to RNA processing has been less extensively characterized. However, available studies indicate that the C-terminal domain participates in coupling transcriptional activation to subsequent steps of gene expression. Early in vitro work showed that PGC‑1α interacts with the Med1 subunit of the Mediator complex, a.k.a. TRAP220 (thyroid hormone receptor-associated protein 220) [75, 78]. The Mediator is a large protein complex comprised of over 20 subunits with diverse roles including transcription initiation, elongation, and termination [79]. During transcription initiation, the Mediator acts as a bridge that links DNA-bound transcription factors to the CTD of RNA polymerase II (RNAPII) [79]. The interaction between PGC‑1α and Med1 maps to amino acids 565–677 of PGC‑1α—overlapping with the RS domains—and to two regions of MED1, one that contains its LXXLL motifs (580–701) and another in the C-terminal region (947–1232), with the latter exhibiting stronger affinity towards PGC‑1α [75, 78].

The importance of the PGC‑1α/Med1 interaction was demonstrated in assays employing histone-free DNA templates [75]. PGC‑1α constructs lacking the C‑terminal region (PGC‑1α 1–505) fail to stimulate transcription in the presence of Med1/Mediator, despite binding to PPARγ. Deletion of the N‑terminal region (PGC-1α 334–797) also leads to failure to activate PPARγ-driven transcription on chromatin templates in the presence of p300 and acetyl-CoA, in this case due to disruption of the chromatin remodeling step required before transcription initiation [75]. This highlights a division of labor in which the PGC‑1α N-terminal domain facilitates chromatin accessibility by recruiting HAT-containing coactivators, while the C‑terminal domain supports productive engagement with RNAPII [75]. Given that Med1 also binds nuclear receptors through LXXLL motifs (same region implied in weaker interactions with PGC‑1α), and that PGC‑1α can directly associate with RNAPII [74], it has been proposed that MED1 contributes to coordinating transfer of PGC‑1α from nuclear receptor–DNA complex to RNAPII for subsequent RNA-processing steps [75].

The RNA-binding capacity of PGC-1α further extends its regulatory scope. In glucagon-stimulated hepatocytes, endogenous PGC-1α associates with numerous mRNAs, including transcripts that do not originate from genes under its canonical transcriptional control [80]. Depletion of PGC-1α reduces the expression of a subset of these transcripts, particularly those induced by glucagon, while leaving others largely unaffected. Notably, Slc25a25—a mitochondrial nucleotide transporter critical for ATP and GTP exchange—is strongly dependent on PGC-1α under these conditions, highlighting an additional role of PGC-1α in sustaining the energetic demands of gluconeogenesis in a manner independent of its transcriptional activation ability [80].

In addition to binding RNA, RS domains contribute directly to mRNA export. PGC-1α interacts directly with the nuclear RNA export receptor NXF1 (nuclear RNA export factor 1) via its RS domains, facilitating the export of transcripts encoding mitochondrial proteins, such as transcription factor A mitochondrial (TFAM) and cytochrome c oxidase (COX) subunits, which are canonical PGC-1α gene targets [81]. Deletion of RS domains leads to nuclear retention of these transcripts without altering their overall expression, indicating that transcriptional activation can be uncoupled from mRNA export [81]. Beyond canonical targets, PGC-1α also regulates the nuclear export of non-canonical transcripts involved in processes such as double-strand DNA repair and telomere maintenance, pointing to a broader role in controlling mRNA localization independently of promoter binding [81].

Amino acids 784–792 (785–793 in humans) of the protein form a conserved sequence also found in PGC-1β, FDSLLKEAQ [61]. This sequence adopts an α-helix capable of binding to the N-terminal region of cap-binding protein 80 (CBP80) within the cap-binding complex (CBC). The CBC is a heterodimer composed of CBP80 and CBP20 that plays diverse roles in RNA metabolism, such as recruiting protein factors that promote transcriptional elongation, facilitating the recognition of splice sites, and stabilizing the 3’ processing complex [61, 82]. The CBC associates with the 5’ cap of pre-mRNAs, a modification that occurs as soon as newly synthesized RNA is extruded from RNAPII and that involves the addition of 7-methylguanosine cap at the 5’ end via reactions catalyzed by enzymes recruited to the RNAPII CTD [82, 83]. While CBP20 directly recognizes the cap structure, CBP80 provides a platform for protein interactions, including binding to the PGC‑1α CPB80-binding motif (CBM) [61]. This interaction enables PGC‑1α to associate with capped pre‑mRNAs and is required for the expression of a large subset (> 75%) of PGC-1α-regulated genes, as demonstrated by reduced transcript levels in cells expressing CBM‑deficient mutants [61].

Beyond protein recruitment, the CBM contributes to the regulation of transcription elongation by facilitating release of RNAPII from promoter‑proximal pausing (PPP) [84]. PPP is a widespread regulatory checkpoint characterized by transient arrest of RNAPII shortly after transcription initiation and is common among genes responsive to signaling inputs [85]. Transition into productive elongation is mediated by the kinase activity of P‑TEFb (positive transcription elongation factor), which phosphorylates RNAPII and associated factors to promote processive RNA synthesis [83, 85]. In contrast, failure to resolve the paused state can lead to premature termination through recruitment of the Integrator complex, which harbors RNA endonuclease capability and can cleave nascent transcripts [86, 87]. Rambout et al. [84] demonstrated the existence of a multiprotein complex comprised of ERRα, PGC‑1α, CBC, Mediator, RNAPII and P‑TEFb, in which PGC‑1α participates in quality control of newly synthesized RNA, promoting elongation provided that pre-mRNAs have been capped and are bound by the CBC. In this model, the complex ERRα/PGC‑1α/CBC/Mediator recruits P‑TEFb during PPP to drive RNAPII release from PPP and simultaneously prevents premature transcription termination by limiting Integrator association [84]. Mediator appears to have a stabilizing role in this complex through interactions with the RS domains of PGC‑1α, and in this context promotes transcription by overcoming PPP rather than augmenting transcription initiation [84]. Importantly, unlike ligand‑activated receptors such as PPARγ, ERRα does not directly bind Mediator, suggesting that PGC‑1α may act as a critical bridging factor in constitutively active transcriptional systems [84].

In parallel, RNA binding appears to contribute to the spatial organization of PGC‑1α within the nucleus. Proteomic analyses indicate that a large fraction (> 80%) of protein-protein interactions involving the PGC‑1α CTD depend on the presence of RNA, with PGC‑1α displaying low binding specificity that enables it to recognize and bind diverse RNA motifs [88]. These RNA-dependent interactions mediated by the RRM were found to be important for the localization of PGC‑1α to chromatin condensates, which are membraneless compartments formed through liquid–liquid phase separation that concentrate transcriptional and RNA‑processing machinery [88]. Therefore, the ability of PGC-1α to bind RNA appears to contribute to the assembly of functional transcriptional complexes within the nucleus.

The functional importance of the CTD is further reflected in the differential gene expression of truncated isoforms lacking RS and RRM domains (PGC‑1α2, −3, −4). These isoforms display distinct transcriptional outputs despite retaining the N‑terminal activation region and part of the repression domain—with hundreds of genes that are uniquely regulated by specific isoforms [89]. The gene specificity of these variants is largely determined by the sequence composition of their N-terminal region, which can also influence alternative splicing despite lacking the CTD via alternative promoter usage [89]. Together, these observations indicate that the CTD expands the regulatory scope of PGC‑1α, enabling coordination of RNA processing, export, and elongation to shape gene expression programs.

### Structure

In terms of three-dimensional structure, experimental evidence suggests that PGC-1α is an intrinsically disordered protein (IDP) [90, 91]. IDPs are proteins that lack a stable, well-defined three-dimensional structure, instead existing as dynamic conformational ensembles that continuously shift within the cellular environment [92]. This structural plasticity is frequently associated with rapid protein turnover, as IDPs can be targeted for degradation by the 20S proteasome through an ubiquitin-independent mechanism known as “degradation by default”, in which the degree of disorder correlates positively with degradation susceptibility [93]. Consistent with this, ectopically expressed PGC-1α (in HEK-293T cells) has a short half-life of approximately 30 min, which is extended when co-expressed with NADH quinone oxidoreductase 1 (NQO1) [90]. NQO1 inhibits substrate degradation by associating physically with the 20S proteasome core particle and restricting substrate access, acting as a gatekeeper whose binding affinity increases in the presence of NADH in a dose-dependent manner [94].

Although protein half‑life can provide indirect insight into structural disorder, it does not constitute definitive evidence. Notably, alternative PGC‑1α isoforms exhibit substantial differences in stability despite sharing extensive sequence identity. The truncated isoform NT‑PGC‑1α (270 amino acids) displays a half‑life exceeding 7 h in HeLa cells [95], while PGC‑1α4 (266 amino acids; residues 13–263 shared with the canonical isoform) has a reported half‑life of approximately 4 h in myotubes [89]. In contrast, PGC‑1α2 and PGC‑1α3—both lacking the C‑terminal domain and portions of the N‑terminal region—exhibit half‑lives comparable to that of full‑length PGC‑1α (approximately 0.5 h in myotubes) [89]. These variations, observed despite high sequence overlap among isoforms, indicate that protein stability is influenced not solely by intrinsic disorder but also by domain architecture, which governs subcellular localization and exposure to compartment‑specific degradation dynamics, as well as the presence or absence of sequence motifs recognized by targeted proteolytic pathways (discussed further in the “Ubiquitination” subsection).

Biophysical analyses of the N-terminal region of PGC-1α (amino acids 2–220) further support the intrinsically disordered nature of this fragment of the protein, characterized by low compactness and a tendency to adopt extended conformations in solution [91]. Docking to transcription factors such as ERRγ triggers a localized disorder-to-order transition that reduces conformational flexibility. Such binding-induced structural rearrangements are thought to enhance structural stability and facilitate the assembly of transcriptional complexes, including recruitment of transcriptional coactivators required for chromatin remodeling [91].

**Fig. 2:**
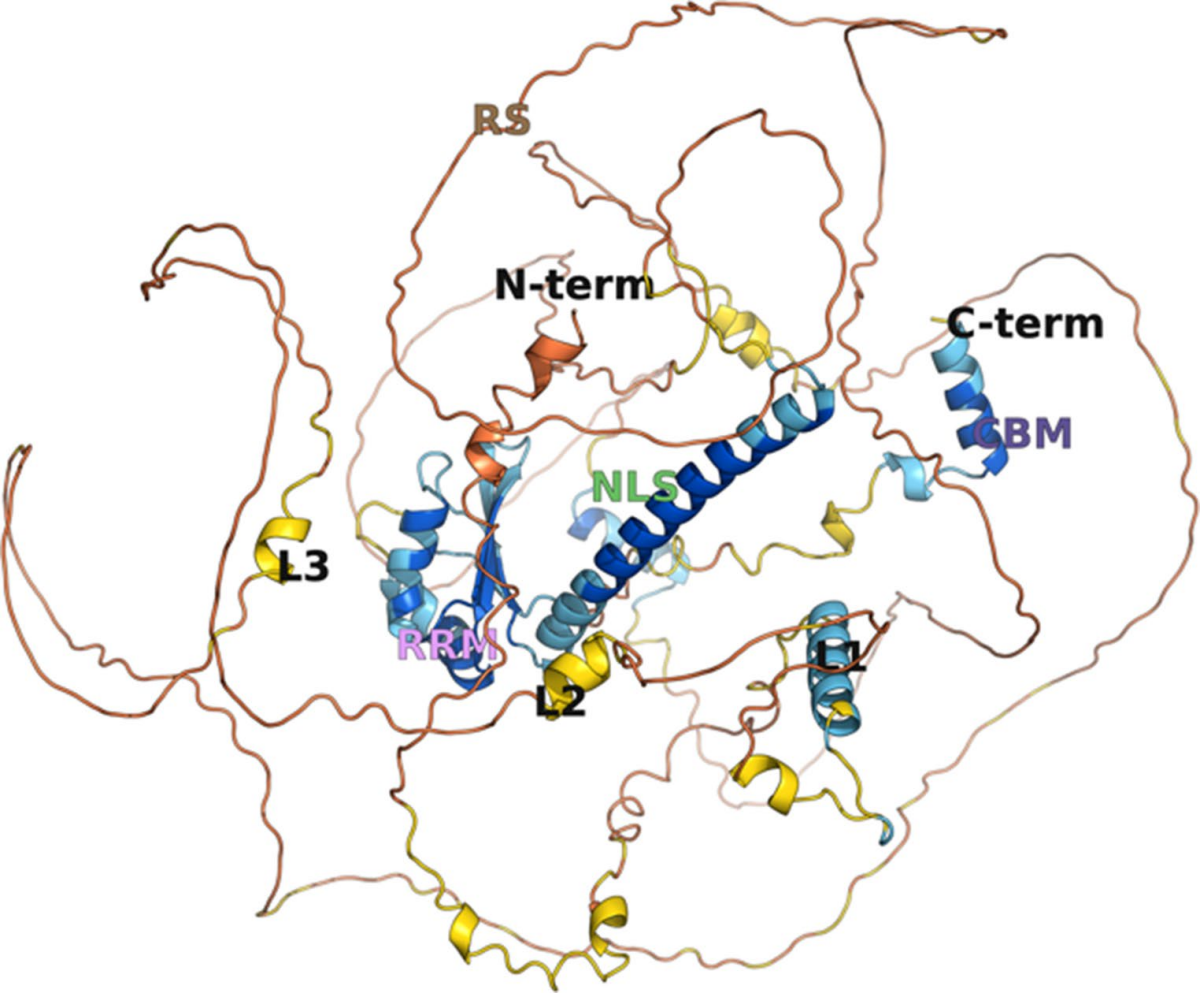
3D structure of the full-length human PGC-1α predicted by AlphaFold2 [96, 97], rendered with PyMOL [98]. Dark blue = very high predicted local distance difference test (pLDDT > 90), light blue = high pLDDT (> 70), yellow = low pLDDT (> 50), orange = very low pLDDT (< 50). The approximate location of some of the domains can be inferred from the proximity to their respective labels

Consistent with experimental data, the AlphaFold2 predicted structure of full-length PGC-1α [96, 97] (Fig. 2) exhibits a low predicted local distance difference test (pLDDT) score. The pLDDT metric reflects the confidence in structural predictions, with values above 90 denoting high accuracy and values below 50 indicating low confidence due to intrinsic disorder. PGC-1α has an average pLDDT of only 52.75, with most regions scoring in the low or very low range. Higher-confidence predictions are predominantly confined to the C-terminal region, where evolutionarily conserved sequences, such as the RRM and CBM, form well-defined secondary structures.

The intrinsically disordered nature of PGC-1α presents a significant challenge for structural characterization with traditional techniques such as X-ray crystallography. Experimentally determined structures of PGC-1α are currently limited to short peptides within the leucine-rich regions bound to nuclear receptor ligand-binding domains [99]. Consequently, recent studies reporting small-molecule docking to ‘PGC-1α’ based on these structures should be interpreted as probing nuclear receptor binding rather than direct interaction with PGC-1α itself.

The conformational flexibility of PGC‑1α has important functional consequences, enabling it to operate as a context‑dependent regulatory scaffold rather than a fixed structural entity. The inherent flexibility of IDPs allows them to bind to various protein partners via low-affinity, dynamic interactions, such that a single protein can participate in multiple regulatory complexes [100]. In the case of PGC‑1α, this property underlies its ability to engage distinct transcription factors across tissues, including PPARγ in brown adipose tissue, HNF4α (hepatocyte nuclear factor 4α) in liver, and MEF2 (myocyte enhancer factor 2) family members in muscle (see “Interactions with transcription factors”) [101], directly influencing gene expression patterns according to available tissue-enriched transcription factors. These interactions are further shaped by post‑translational modifications, which can alter binding affinities, subcellular distribution, and protein stability in response to metabolic signals (e.g., fasting in the liver, β_3_-adrenergic signaling in brown adipose tissue [101]).

Additional layers of specificity arise from isoform diversity. Some PGC‑1α transcripts generated through alternative promoter usage or alternative splicing exhibit tissue‑restricted expression patterns, including liver [102], brain [10, 11], and muscle‑specific isoforms [103]. These tissue-specific exons frequently map to intrinsically disordered regions enriched in post‑translational modification sites, which can reshape interaction networks according to cellular environment [104]. Together, these features indicate that the functional output of PGC‑1α is not determined solely by its primary sequence, but by the interplay between structural flexibility, binding partner availability, and regulatory modifications within specific tissues. This organization provides a mechanistic basis for the context‑dependent and often divergent physiological effects attributed to PGC‑1α.

## Interactions with transcription factors

In general, transcription factors are proteins that bind to DNA as monomers, hetero- or homodimers to regulate gene expression [7]. Nuclear receptors are a class of transcription factors whose activity is modulated by the binding of small molecule ligands, such as hormones or metabolites, which dock to the ligand binding pocket (LBP) within the ligand binding domain (LBD) of the receptor. The docking event induces conformational changes that lead to the recruitment of transcription coactivators, which typically interact with the LBD via their LXXLL motif [105]. This interaction is further stabilized by the formation of a “charge clamp pocket” involving two conserved residues that interact with the LXXLL motif: a glutamate residue in the activating function-2 (AF-2) helix and a lysine residue in the helix 3 (H3) [106].

**Fig. 3:**
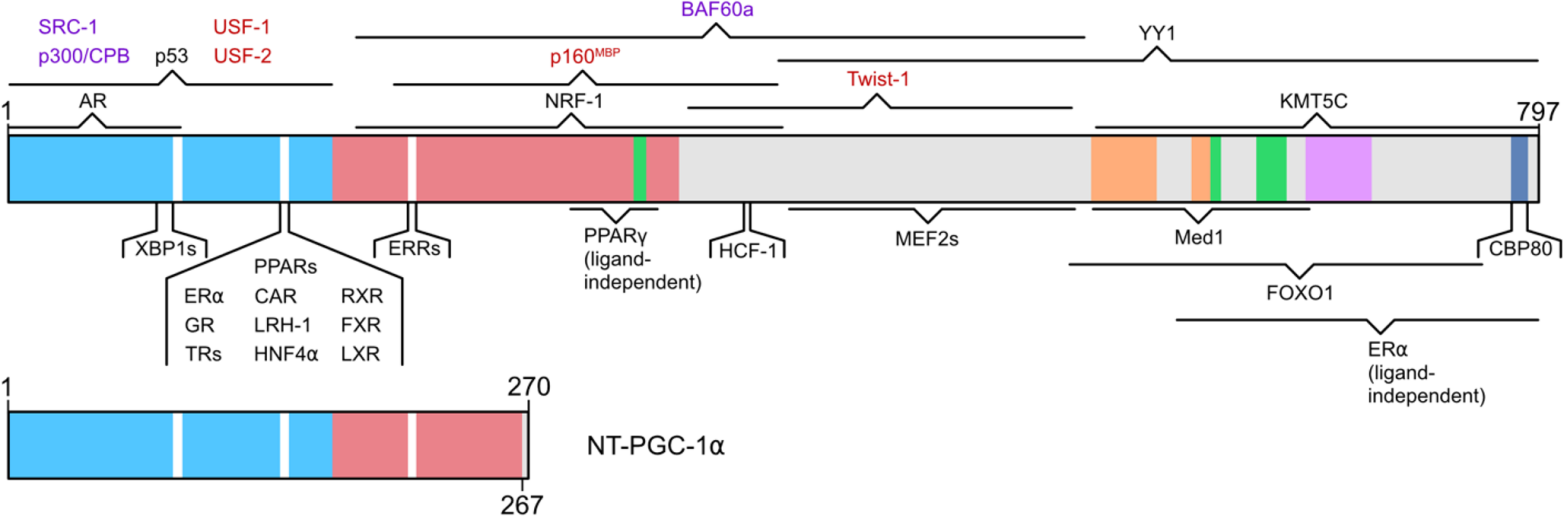
Key binding sites on full-length murine PGC-1α for several proteins (chromatin remodelers in purple, repressors in red, and remaining partners in black). NT-PGC-1α is shown below full-length PGC-1α for comparison

PGC-1α interacts with nuclear receptors through both ligand-dependent and ligand-independent mechanisms. The former follows the typical LXXLL-mediated binding, while the latter varies depending on the receptor [6]. This section provides an overview of the key transcription factors that associate with PGC-1α, detailing the binding domains involved, and the biological consequences of PGC-1α-mediated coactivation. A graphical summary of the binding regions for various PGC-1α partners is provided in Fig. 3. The domains of the alternative splicing truncated PGC-1α isoform NT-PGC-1α are also shown alongside the full-length protein for comparison.

### Peroxisome proliferator-activated receptors (PPARs)

PPARα, PPARβ/δ and PPARγ form a subgroup of nuclear receptors that undergo heterodimerization with the retinoid X receptor (RXR) and recruitment of coactivators upon binding of activating ligands [107], which are typically fatty acids and their derivatives [25]. The isoforms differ with regards to tissue distribution, ligand specificities, and physiological function, exhibiting some degree of overlap in activity [108].

### PPARγ

PPARγ is primarily expressed in brown and white adipose tissue, where it regulates adipocyte differentiation, lipid storage, adipogenesis (lipid deposition), and energy balance [108]. In brown fat, PGC-1α coactivates PPARγ to enhance UCP1 expression in response to stimuli such as cold exposure or β-adrenergic receptor activation [6]. Although white adipose tissue naturally has low levels of PGC-1α, ectopic expression of PGC-1α increases UCP1 levels—a response that is further amplified by treatment with the synthetic PPARγ agonist rosiglitazone [109]. Additionally, treated cells exhibit increased mRNA levels of respiratory chain proteins and fatty acid oxidation enzymes, acquiring a phenotype akin to brown adipocytes [109].

The ligand-independent PPARγ-binding domain of PGC-1α was identified as a proline-rich sequence spanning amino acids 292–338, while residues 181–505 of PPARγ were found to be involved in this interaction [6]. Later, crystallization studies revealed that the L2 motif of PGC-1α forms an α-helix that binds with high affinity and specificity to the LBD of PPARγ bound to rosiglitazone (PDB ID: 3CS8) [110]. Mutating this LXXLL motif significantly reduces binding affinity, thereby weakening PGC-1α-mediated coactivation, both in the presence and absence of the agonist [110]. Additional X-ray diffraction studies confirmed the strong affinity of the LXXLL motif for PPARγ in both agonist-bound [111, 112] and ligand-free states [113].

### PPARα

PPARα is highly expressed in energy-demanding tissues, such as the heart, liver, skeletal muscle and BAT, where it regulates genes involved in fatty acid metabolism [108]. As such, coactivation of PPARα by PGC-1α leads to the upregulation of key mitochondrial fatty acid oxidation enzymes (e.g., medium-chain acyl-CoA dehydrogenase (MCAD), long-chain acyl-CoA dehydrogenase (LCAD), carnitine palmitoyltransferase 1 (CPT1)) and enhances fatty acid oxidation rates [114]. In brown adipocytes, ligand-activated PPARα binds to the PPAR-responsive element (PPRE) in the PGC-1α gene promoter, inducing PGC-1α expression [115]. Similar to PPARγ, PPARα coactivated by PGC-1α upregulates UCP1 in brown adipocytes, with a 40-fold increase observed when treated with the synthetic PPARα agonist Wy 14,643 [116].

The L2 motif of PGC-1α and the AF-2 region of PPARα are required for this interaction [114]. X-ray diffraction studies confirmed this mechanism by solving the structure of a human PGC-1α peptide (amino acids 135–156) containing the L2 motif docked to PPARα bound to a synthetic agonist [117].

### PPARβ/δ

PPARβ, also known as PPARδ, is the least studied among the three PPAR isoforms and is ubiquitously expressed, with higher levels in the liver, adipose tissue and skeletal muscle [108]. Some of its roles overlap with PPARα: for example, in brown adipocytes and myotubes, activation of PPARβ by the synthetic agonist GW501516 induces the expression of mitochondrial uncoupling proteins and fatty acid oxidation enzymes, leading to an increase in fatty acid oxidation rates [118, 119].

PGC-1α coactivates PPARβ even in the absence of an agonist, and like other PPARs, this interaction is mediated by the PGC-1α LXXLL motif [118]. Mutation of this motif to LXXAA completely disrupts the interaction with ligand-bound PPARβ [118]. In vascular endothelial cells, coactivation of ligand-bound PPARβ by PGC-1α promotes the expression of heme oxygenase-1 (HO-1), an enzyme with anti-inflammatory, anti-apoptotic and antioxidant properties, aiding in the protection against atherosclerosis [120]. In addition, PPARβ may have a protective role against PGC-1α degradation, as overexpression of PPARβ was shown to increase PGC-1α protein levels by protecting it from ubiquitination and subsequent degradation by the ubiquitin-proteasome system [121].

### Estrogen-related receptors (ERRs)

Estrogen-related receptors (ERRs) are a subfamily of nuclear hormone receptors that include three isotypes: ERRα, ERRβ, and ERRγ. They share sequence similarity with estrogen receptors (ERs), but unlike traditional ERs, do not bind estrogens [122]. Instead, ERRs are classified as orphan receptors due to the absence of identified endogenous ligands [122]. They exhibit low basal transcriptional activity, which is potentiated in a ligand-independent manner by coactivators, such as PGC-1α [123]. ERRs function as obligate homodimers for transcriptional activation [124], and under physiological conditions PGC-1α binds to only one of the homodimer subunits [125].

ERRα and ERRγ are coexpressed with PGC-1α in metabolically demanding, mitochondria-rich tissues, e.g., heart, kidneys, BAT [122]. Genomic studies reveal a considerable overlap between ERRα and ERRγ target genes, with 63–84% ERRα genes also being targeted by ERRγ in the mouse heart [126]. In cardiac myocytes, the PGC-1α/ERRα complex upregulates genes involved in fatty acid uptake, oxidation, and mitochondrial respiration, an effect that is partly mediated indirectly through increased PPARα transcription [127]. In skeletal muscle cells, overexpression of PGC-1α/β enhances protein synthesis and myotube growth via an ERRα-dependent pathway [128]. Moreover, PGC-1α induces ERRα expression [129], and ERRα expression is crucial for PGC-1α to induce mitochondrial biogenesis genes, including TFAM (mitochondrial transcription factor A) and Tim22 (translocase of the inner mitochondrial membrane 22) [130].

In hepatocytes, ERRγ is coactivated by PGC-1α in response to glucagon signaling, regulating the expression of key gluconeogenesis enzymes phosphoenolpyruvate carboxykinase 1 (PCK1) and glucose-6-phosphatase (G6PC) [131]. This effect is reduced by treatment of cells with GSK5182, a selective ERRγ inverse agonist that disrupts PGC-1α binding to ERRγ without affecting the DNA binding ability of the receptor [131]. However, compared to other isotypes, ERRγ transcriptional activity is generally less dependent on coactivation by PGC-1α/β [132]. For example, in brown adipocytes, overexpression of ERRγ promotes UCP1 expression even in the absence of PGC-1α [133].

Unlike PPARs, the LXXLL (L2) motif of PGC-1α is not the main interaction site for ERRs, acting instead as an accessory low affinity binding site [65]. The LLKYL (L3) motif is the primary binding site, as the removal of this sequence results in an almost complete loss of ERRα coactivation [65]. The much less studied ERRβ isoform has several roles in tissue development, including the placenta, inner ear, and retina [134]. Crystallography studies show that, like the other ERR isotypes, ERRβ exhibits strong selectivity for the PGC-1α LLKYL motif (PDB ID: 6LN4) [135].

### Hormone receptors

#### Estrogen receptor α (ERα)

In the absence of a ligand, ERα primarily resides in the nucleus, shuttling between the nucleus and the cytoplasm by passive diffusion and active transport [136]. Upon binding of an agonist, the receptor undergoes a conformational change leading to dimerization and further accumulation in the nucleus [136, 137]. The dimeric complex can then recognize estrogen response elements (EREs) in the regulatory regions of target genes to modulate their expression [138].

Full-length PGC-1α can bind to ERα even in the absence of a ligand. This interaction is enhanced in the presence of an agonist (17β-estradiol) but remains unaffected by an antagonist (tamoxifen). Ligand-independent binding occurs within the C-terminal half of PGC-1α (amino acids 604–797) and is mediated by the hinge region of ERα (amino acids 253–283) [138]. In contrast, ligand-dependent binding involves the LXXLL motif of PGC-1α and the AF-2 domain of ERα. Upon agonist binding, ERα undergoes conformational changes that create a hydrophobic cleft in the AF-2 domain, allowing the PGC-1α LXXLL motif to dock [138].

In addition to coactivating ERα, PGC-1α transcription was demonstrated to be under ERα control in the hearts of female mice, with ligand-bound ERα enhancing PGC-1α promoter activity [139]. Consistently, in adult cardiomyocytes, treatment with 17β-estradiol potently activates PGC-1α expression and leads to upregulation of mitochondrial biogenesis and respiratory chain genes, such as TFAM and COX subunit 1 (COX1), respectively [140]. This effect is also evident in pathological conditions such as cardiac traumatic hemorrhage, where administration of 17β-estradiol was found to increase PGC-1α protein levels by approximately 20%, suggesting a cardioprotective effect [141].

#### Glucocorticoid receptor (GR)

The glucocorticoid receptor (GR) primarily resides in the cytoplasm, where it forms a complex with heat shock proteins (HSPs) that mask its nuclear localization signals. Upon ligand binding, GR dissociates from HSPs and translocates to the nucleus, where it recognizes and binds to glucocorticoid response elements (GREs) [142]. GR binds both endogenous ligands, such as hydrocortisone—a glucocorticoid derived from cholesterol secreted by the adrenal gland—and synthetic glucocorticoids, such as dexamethasone, which are used to treat inflammatory conditions, allergic reactions, and autoimmune diseases [143].

Beyond maintaining homeostasis, endogenous glucocorticoids play a crucial role in fetal tissue and organ maturation, particularly in the lungs and heart. A physiological rise in glucocorticoid levels before birth is essential for neonatal survival [144]. In the fetal heart, studies in primary mouse cardiomyocytes have shown that glucocorticoid activation of GR increases PGC-1α mRNA expression in a dose-dependent manner. This upregulation of PGC-1α is necessary for cardiomyocyte development, affecting sarcomere length, Z-disc width, and mitochondrial function [145].

High-resolution crystallographic analysis of PGC-1α bound to an ancestral variant of GR (AncGR2) revealed that the PGC-1α LXXLL motif docks into a hydrophobic groove formed by the AF-2 domain of AncGR2, where it interacts with leucine residues through hydrophobic contacts [146]. This interaction is further stabilized by an electrostatic bond between Asp59 and Lys145 in PGC-1α, with the latter Lys residue being located in the center of the LXXLL motif [146]. The dexamethasone-bound receptor exhibits a more stable structure compared to hydrocortisone, leading to enhanced PGC-1α recruitment [146].

#### Androgen receptor (AR)

The androgen receptor (AR) resides in the cytoplasm and translocates to the nucleus in a manner similar to GR. Its physiological ligands include testosterone and dihydrotestosterone (DHT). Once in the nucleus, agonist-bound AR binds to androgen response elements (AREs) in the promoter region of target genes. Beyond its role in male phenotype development and maintenance, AR is also implicated in the pathogenesis of prostate cancer [147].

Specifically, enhanced AR activation mediated by transcriptional coactivators is thought to contribute to castration-resistant prostate cancer [148]. In this context, AR has been shown to interact with PGC-1α both in vitro and in vivo, and this interaction is independent of DHT binding. The N-terminal region of PGC-1α (at least residues 1–89, which only partially include the L1 motif) is involved in binding to the N-terminal transactivation domain of AR, consistent with the ligand-independent nature of this interaction. PGC-1α coactivation promotes AR homodimerization, enhancing ARE binding and upregulating target gene expression, which includes prostate-specific antigen (PSA), frequently found in elevated levels in prostate cancer [148].

#### Thyroid hormone receptors (TRs)

Thyroid hormone receptors mediate the effects of thyroid hormone (T3, triiodothyronine) by binding to thyroid hormone response elements (TREs) in target genes, usually as heterodimers with retinoid X receptors (RXRs). TRs are often maintained in a repressive state through association with corepressors with histone deacetylase activity, and become transcriptionally active upon ligand binding, which promotes the recruitment of coactivators such as PGC-1α [149]. For instance, in the liver, PGC-1α coactivation of T3-bound TRβ leads to upregulation of CPT-1α, the rate-limiting enzyme of mitochondrial fatty acid oxidation [150].

PGC-1α enhances the transcriptional activity of all transcriptionally active TR isoforms (TRα1, TRβ1, and TRβ2). The interaction with TRβ1 is largely ligand-dependent and mediated by the PGC-1α LXXLL motif and the AF-2 domain of the receptor, with the integrity of the helix 1 also being required for the interaction [149]. However, evidence suggests that TRβ1 can also bind PGC-1α independently of a ligand through its N-terminal AF-1 domain, which interacts with the N-terminal activation region of PGC-1α (amino acids 1–130). This ligand-independent interaction is proposed to facilitate initial recruitment of PGC-1α to promoter regions of target genes prior to full receptor activation [151].

Within the PGC-1α gene, a TRE located upstream of the transcriptional start site allows TRα1 binding [152]. Accordingly, administration of T3 was shown to lead to increased PGC-1α mRNA and protein levels in hepatocytes, suggesting PGC-1α can regulate its own expression [152]. In addition, PGC-1α influences TR signaling at a post-transcriptional level by modulating the splicing of TR isoforms. Specifically, PGC-1α shifts the TRα1:TRα2 ratio in favor of TRα2, a variant that does not bind T3 and acts as a negative regulator of other TR isoforms [153]. This effect requires the C-terminal region of PGC-1α containing the RRM, providing direct evidence that RNA-processing functions can impact endogenously expressed genes [74]. These observations support the existence of an autoregulatory loop in which T3 induces PGC-1α expression, PGC-1α enhances TR-mediated transcription [152], and RNA-level regulation contributes to attenuation of prolonged thyroid hormone signaling [153].

**Fig. 4:**
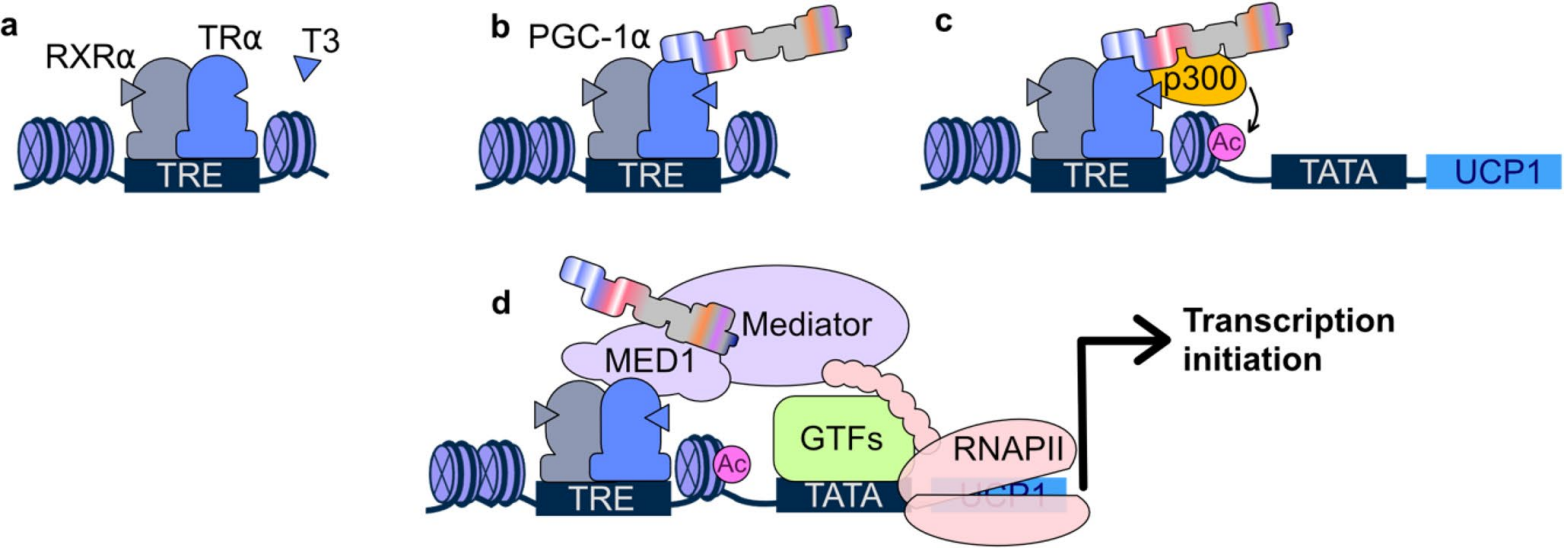
Schematic four-panel diagram showing how PGC-1α participates in the assembly of transcriptional complexes according to the model proposed by Chen et al. [78]: **a** TRE-bound TRα/RXRα heterodimer is inactive due to absence of the ligand (T3); activated TRα binds PGC-1α (gradient-colored domains approximately following Figs. 1 and 3); **c** PGC-1α recruits chromatin remodeler p300 which performs histone acetylation to expose the UCP1 promoter region; **d** Mediator is recruited to the TRα/RXRα/PGC-1α complex, with the MED1 subunit displacing the TRα/PGC-1α interaction and binding to PGC-1α RS region. In parallel, general transcription factors (GTFs) are recruited to the exposed promoter as well as RNAPII. Mediator associates with RNAPII to stabilize the pre-initiation complex

In addition to transcriptional and RNA-mediated mechanisms, the C-terminal region of PGC-1α contributes to the assembly of transcriptional complexes under TR control. The UCP1 gene contains an enhancer region recognized by TRα/RXRα heterodimers, which can bind in a ligand-independent manner; the presence of T3 is required for recruitment of both PGC-1α and the Mediator complex [78]. In the mechanistic model proposed by Chen et al. [78] and depicted in Fig. 4, PGC-1α is initially recruited through LXXLL-mediated interactions with TRα, and promotes chromatin accessibility by recruiting remodelers such as p300, SRC-1, and BAF60a. Interactions with TRα are eventually displaced by competition with MED1, yet PGC-1α remains associated with the complex through stronger interactions with the C-terminal region of MED1 (947–1232) via its RS domains (551–677) [78]. This ligand-dependent cooperative binding mechanism results in enhanced recruitment of PGC-1α to the enhancer-bound complex in the presence of Mediator [78] and may translate into increased transcriptional output.

Importantly, this model extends PGC-1α function beyond simple nuclear receptor coactivation: PGC‑1α participates in the dynamic reorganization of transcriptional complexes, transitioning from receptor-bound states at enhancers to Mediator-associated assemblies linked to RNAPII. The RNA-related activities discussed earlier suggest that additional functional states emerge after transcription initiation, in which PGC‑1α can interact with RNAPII, the 5’ cap of nascent transcripts, and other components of the transcriptional machinery. Within this framework, PGC‑1α contributes to multiple stages of gene expression under TR control, including chromatin remodeling, formation and progression of the transcriptional complex, and regulation of TR isoform splicing. However, in contrast to transcriptional mechanisms, which are supported by detailed biochemical models, the contribution of PGC‑1α to alternative splicing remains less well defined. While the requirement for the C‑terminal region and RRM suggests involvement in RNA recognition or recruitment of RNA-processing factors, the molecular determinants governing splice-site selection and spliceosome engagement have not been established. Consequently, although PGC‑1α clearly influences TR isoform composition, the mechanisms by which it links transcriptional activation to splicing decisions remain unresolved.

### Xenobiotic receptors

Xenobiotic receptors act as sensors for toxic endogenous and exogenous compounds, regulating the transcription of drug-metabolizing enzymes and transporters to facilitate the elimination of harmful chemicals from the body [154]. Among these receptors, pregnane X receptor (PXR) and constitutive androstane receptor (CAR) are known for their broad xenobiotic activation and overlapping roles in the liver and intestine, where they regulate many of the same xenobiotic-metabolizing genes [154].

In mouse liver, PXR and CAR are retained in the cytoplasm through complexes with heat shock proteins (HSP90) and cytoplasmic CAR retention protein (CCRP) [155, 156]. Upon ligand activation, these receptors dissociate from their complexes, translocate to the nucleus, and form heterodimers with RXR [157]. Both PXR/RXR and CAR/RXR heterodimers are able to recognize various response elements in promoter regions, including direct repeat elements (DR-3, DR-4) and everted repeat elements (ER-6, ER-8), to regulate the transcription of their target genes [158, 159].

#### Pregnane X receptor (PXR)

The pregnane X receptor (PXR) was originally named for its ability to bind natural and synthetic C21 sterols (pregnanes) and can be activated by antibiotics, environmental pollutants, and natural steroids [158]. PGC-1α overexpression in hepatocytes upregulates PXR through PGC-1α-mediated coactivation PPARα [160], as PXR expression is under PPARα transcriptional control [161]. Additionally, PGC-1α directly coactivates PXR, enhancing PXR-induced transcription of the drug-metabolizing enzyme cytochrome P450 3A11 (CYP3A11) in the presence of the agonist pregnenolone-16α-carbonitrile [160].

Beyond its role in drug metabolism, PXR binds toxic bile acids such as lithocholic acid and represses the transcription of cholesterol-7α-hydroxylase (CYP7A1), the first enzyme in bile acid synthesis from cholesterol [162]. Corroborating with this, it was found that ligand-activated PXR suppresses bile acid production in hepatocytes [163]. The proposed mechanism of suppression involves PXR binding PGC-1α and competing with HNF4α—a nuclear receptor that activates bile acid biosynthesis genes, including CYP7A1—for interaction with PGC-1α [163]. This interaction is ligand-dependent and mediated by the LBD of PXR [163].

#### Constitutive androstane receptor (CAR)

CAR exhibits constitutive activity in immortalized cell lines such as HepG2, where it accumulates in the nucleus even in the absence of a ligand [164]. This activity is suppressed by the inverse agonists androstanol and androstenol, which promote coactivator release from the CAR LBD [165]. However, in primary hepatocytes and intact liver in vivo, CAR is predominantly cytoplasmic and translocates to the nucleus when induced by a xenobiotic agent such as the anticonvulsant drug phenobarbital [156, 166].

Transient transfection studies showed that PGC-1α strongly enhances CAR activity even in the absence of a ligand, while PGC-1β has a weaker effect [167]. CAR/RXR heterodimerization stabilizes PGC-1α binding, augmenting the PGC-1α-mediated CAR activation [167]. The interaction between PGC-1α and CAR is mediated by the LXXLL motif and RS domains of PGC-1α [167], as well as conserved glutamate and lysine residues in AF-2 and H3 of the CAR LBD, respectively, as mutations at these residues disrupt the interaction [168].

In addition to its role in xenobiotic detoxification, CAR regulates energy metabolism by suppressing gluconeogenic and fatty acid oxidation genes, which are normally upregulated by HNF4α [169]. CAR competes with HNF4α for binding to the DR-1 motif in the promoter region of gluconeogenic genes such as PCK1 [169], and also competes with HNF4α for binding to common coactivators such as PGC-1α, with the latter effect being enhanced by CAR agonists [169]. Moreover, ligand-activated CAR promotes PGC-1α sequestration into promyelocytic leukemia protein-nuclear bodies (PML-NBs) [168]. PML-NBs are macromolecular nuclear structures that sequestrate proteins and mediate post-translational modifications in response to cellular stresses [170]. In these structures, CAR associates with the Cullin1 E3 ligase, and when complexed with PGC-1α, promotes PGC-1α ubiquitination and subsequent degradation [168]. It is hypothesized that the negative regulatory effect of CAR over PGC-1α represents a cellular adaptation for energy-restricted conditions [168].

### Other Nuclear Receptors

#### Retinoid X receptors (RXRs)

RXRs reside in the nucleus, where they can form either homodimers or heterodimers with various nuclear receptors, including PPARs. In the latter case, RXRs are essential for the efficient DNA binding of their partner receptors, facilitating transcriptional regulation [171, 172]. There are three RXR subtypes—RXRα, RXRβ, and RXRγ—encoded by different genes. While these subtypes share structural homology, their tissue distribution varies. RXRα is predominantly expressed in the liver and kidneys, RXRγ is found in muscle, and RXRβ is ubiquitously expressed; thus, there exists some overlap with PGC-1α expression [171, 172]. RXRs bind to vitamin A metabolites, exhibiting a particularly high affinity for 9-cis-retinoic acid [171].

PGC-1α has been shown to enhance ligand-stimulated RXR transcription, as demonstrated in gene transfection assays. This effect is observed across all three RXR subtypes and is mediated by the LXXLL motif of PGC-1α and the AF-2 region of RXR [173]. Recent findings indicate that the specific structure of the ligand bound to RXRα significantly influences coactivator recruitment, particularly in the case of PGC-1α. Variations in ligand interactions with helix 11 of RXRα likely affect the positioning of helix 12 (AF-2), thereby altering the shape and size of the coactivator binding site [174]. The precise physiological role of PGC-1α recruitment to RXRs remains unclear; however, since RXR homodimers can activate PPAR target genes in vivo by binding to DR-1 elements [175], ligand-induced PGC-1α interaction may contribute to promoter-specific effects, such as thermogenic regulation in adipose tissue [174].

#### Liver X receptors (LXRs)

Liver X receptors (LXRs) were initially classified as orphan nuclear receptors until it was discovered that they bind and are activated by mono-oxidized cholesterol derivatives, known as oxysterols. Among these, 22(R)-hydroxycholesterol is one of the most potent endogenous activators [176]. There are two LXR isoforms, LXRα and LXRβ, which share a high degree of amino acid similarity but differ in tissue distribution. LXRβ is ubiquitously expressed, whereas LXRα is specifically abundant in the liver [176].

LXRs regulate gene transcription by forming obligate heterodimers with RXR. These LXR/RXR heterodimers bind to LXR response elements (LXREs) in the promoter regions of target genes [176]. In the absence of a ligand, the heterodimer remains inactive by interacting with transcriptional corepressors [177]. Upon ligand binding, a conformational change occurs that releases corepressors and facilitates the recruitment of coactivators, initiating gene transcription [178, 179]. Overall, LXRs play a crucial role in regulating the expression of genes involved in cholesterol transport and metabolism [180].

PGC-1α has been identified as a ligand-dependent coactivator of LXR, a process that requires the integrity of the LXXLL motif. To achieve maximal transcriptional activation of LXRα, the presence of both an LXR ligand, such as 22(R)-hydroxycholesterol, and an RXR ligand, such as 9-cis-retinoic acid, is necessary [181].

#### Farsenoid X receptor (FXR)

The farnesoid X receptor (FXR), along with PXR and the vitamin D receptor, functions as a bile acid receptor [182]. Humans have only one functional FXR gene, FXRα, which can originate four isoforms (FXRα 1–4) [183]. FXRα is mainly expressed in the liver, small intestine, and kidneys, where it regulates the expression of genes involved in bile acid and lipoprotein metabolism [183]. The receptor is activated by primary bile acids (cholic acid, chenodeoxycholic acid) and secondary bile acids (litholic acid, deoxycholic acid), derived directly from cholesterol and primary bile acids, respectively [184]. Upon activation, FXRα forms an heterodimer with RXR and binds to FXR response elements (FXREs) [184]. The heterodimerization process leads to conformational changes that enhance the binding affinity of the LBD for LXXLL motifs in transcriptional coactivators [185].

Zhang et al. [186] found that ectopic expression of PGC-1α in hepatocytes induces the transcription of FXRα, specifically the isoforms FXRα3 and FXRα4 [186]. This is likely achieved through the coactivation of PPARγ or HNF4α, which can bind to the DR-1 element in the FXRα gene [186]. They also reported that PGC-1α coactivates FXR in an unusual ligand-independent manner that does not involve the LXXLL motif of PGC-1α and is mediated by the FXR DNA binding domain [186]. Contrasting these results, Kanaya et al. [187] and Sakvur et al. [188] independently showed that the interaction between PGC-1α and FXR is ligand-dependent, requiring the PGC-1α LXXLL motif and the FXR LBD (amino acids 226–476). Moreover, mutation of either of the conserved charge clamp residues (E471 and K307) leads to a loss in the ability of PGC-1α to coactivate the receptor [188]. This mode of interaction was confirmed by co-crystallization of ligand-bound FXR with a PGC-1α fragment containing the L2 motif (PDB ID: 5Q0I) [189]. The discrepancies between studies may have been caused by differences in receptor used [188] (mouse for Zhang et al. [186] vs. human [187, 188]) or insufficient ligand concentration for the LBD interaction to take place [187].

#### Liver receptor homolog 1 (LRH-1)

LRH-1 is an orphan nuclear receptor expressed in endoderm-derived tissues, including the intestine, liver, and exocrine pancreas [190]. In these tissues, LRH-1 participates in the regulation of bile acid homeostasis, reverse cholesterol transport, and steroidogenesis [191]. Unlike most nuclear receptors, LRH-1 binds to DNA as a monomer, recognizing the consensus site YCA AGG YCR, where Y represents pyrimidines and R purines [192]. LRH-1 activity is linked to certain types of cancer, where it is implicated in cell proliferation and metastasis [191].

In human breast preadipocytes, LRH-1 is a specific transcriptional activator of the aromatase gene [193]. PGC-1α, which is also expressed in preadipocytes, coactivates LRH-1 and enhances its activity on the aromatase promoter [193]. Increased aromatase expression can promote local estrogen biosynthesis, with estrogen playing a critical role in the development and progression of breast cancer by acting on ERs [194].

In hepatocytes, where LRH-1 is abundantly expressed, the PGC-1α-mediated coactivation of LRH-1 results in upregulation of cholesterol-7α-hydroxylase [195]. The PGC-1α/LRH-1 interaction can be disrupted by the small heterodimer partner (SHP) protein, which is induced by FXR and binds to LRH-1 with high affinity [195]. As with most nuclear receptors, the PGC-1α/LRH-1 interaction is mediated by the LXXLL motif of PGC-1α [193], and the AF-2 domain of LRH-1 [195], a mechanism confirmed by crystallography studies [196]. Notably, the closely related PGC-1α homolog, PGC-1β, does not coactivate LRH-1 [193, 195].

#### Hepatocyte nuclear factor 4α (HNF4α)

HNF4α is a constitutively active nuclear receptor highly expressed in the liver, kidney, intestine, and pancreas [197, 198]. It binds DNA as a homodimer [199], recognizing DR-1 response elements [200]. HNF4α plays key roles in hepatocyte differentiation, liver architecture development [201], and the regulation of glucose, fatty acid, and cholesterol metabolism in the liver [197]. Crystallographic studies of the HNF4α/γ LBDs have revealed the presence of tightly bound endogenous fatty acids in the LBP [202, 203]. These fatty acids are thought to be captured during the protein’s translation and folding process, becoming trapped in its fully folded structure [203].

PGC-1α coactivates HNF4α in the absence of any external ligand, leading to the upregulation of gluconeogenic genes such as PCK1 [27]. The ability of PGC-1α to activate these genes is lost in the absence of HNF4α, though fatty acid β-oxidation genes remain inducible, likely through other transcription factors such as PPARα and ERRα [204]. In stem cells, coexpression of PGC-1α and HNF4α strongly induces the mRNAs of apolipoproteins A-IV and C-II, which are involved in VLDL metabolism, suggesting a role in the regulation of hepatic lipoprotein export [205].

The physical association between PGC-1α and HNF4α is primarily mediated by the LXXLL motif of PGC-1α and the AF-2 domain of HNF4α [27]. This was confirmed through co-crystallization of a PGC-1α peptide bound to the HNF4α LBD [206]. Structural and mutational analyses suggest that multiple leucine-rich motifs in PGC-1α contribute to HNF4α transactivation, as mutations in any of the L1–L3 motifs reduce transcriptional activation, particularly in L2 and L3 [206]. Additionally, mutational studies on HNF4α have identified residues M182, L219, L220, and I338 as critical for enabling PGC-1α coactivation [207].

### Other Transcription Factors

#### Nuclear respiratory factors (NRFs)

Nuclear respiratory factors (NRF-1 and NRF-2) are transcription factors that regulate numerous genes involved in mitochondrial function, including those encoding respiratory chain subunits and factors for mitochondrial DNA transcription and replication [208]. NRF-1 functions as a homodimer that binds DNA directly [209], whereas NRF-2 forms a multimeric complex composed of a DNA-binding α subunit and several regulatory subunits (β₁, β₂, γ₁, γ₂) that associate in different combinations [210].

PGC-1α coactivates NRF-1, enhancing its transcriptional activity on key mitochondrial genes, such as TFAM [211] and the mitochondrial transcription factor B isoforms TFB1M and TFB2M [212]. The interaction between PGC-1α and NRF-1 is mediated by amino acids 180–403 of PGC-1α, which overlap with its PPARγ ligand-independent binding domain, and amino acids 108–143 of NRF-1, which include part of its DNA-binding domain [211]. In addition to promoting mitochondrial biogenesis, the PGC-1α/NRF-1 pathway upregulates the mitophagy receptor FUNDC1, which facilitates the removal of excess or damaged mitochondria [213]. This coordination between mitochondrial biogenesis and mitophagy helps maintain a balanced mitochondrial population, preventing cellular overcrowding [213].

While PGC-1α can transactivate promoters of NRF-2 target genes, such as TFB1M and TFB2M [212], in vitro experiments have shown that PGC-1α does not bind NRF-2α or β subunits directly [214]. Instead, evidence suggests these proteins exist in a complex with host cell factor-1 (HCF-1) [214], a transcriptional cofactor that binds PGC-1α by recognizing the motif DHDY (amino acids 382–385) [2], and that also binds to the β subunit of NRF-2, acting as a coactivator [215]. The formation of such a ternary complex in vivo was demonstrated by Vercauteren et al. [214] for PRC, which shares the HCF-1 binding motif with PGC-1α. Thus, it is likely that similarly to PRC, PGC-1α transactivation occurs through the formation of a complex mediated by HCF-1.

Beyond coactivation, PGC-1α is implicated in the transcriptional regulation of NRF-1 and NRF-2 genes. Ectopic expression of PGC-1α in myotubes and myoblasts was shown to yield a significant increase in NRF-1 and NRF-2α mRNA levels [211], although a later study found only minimal changes in NRF-1 transcript levels in myoblasts overexpressing PGC-1α [212]. A possible explanation for the observed discrepancy between the two studies is that NRF-1 expression may require a longer time to become detectable. Mootha et al. [216] proposed a model where increased PGC-1α levels first induce the expression of ERRα and NRF-2α, which then cooperate with PGC-1α in a double positive feedback loop to amplify their own expression. Once these transcription factors are upregulated, they are coactivated by PGC-1α to drive the expression of NRF-1 and other mitochondrial target genes [216].

#### Foxhead box protein O1 (FOXO1)

FOXO1 is a member of the FOXO transcription factor subfamily, playing key roles in energy homeostasis. It promotes gluconeogenic enzyme expression in the liver during fasting [217] and contributes to the differentiation of pre-adipocytes, myoblasts, and pancreatic β-cells [218]. Insulin signaling represses FOXO1 activity through phosphorylation by Akt, a serine/threonine kinase downstream of the insulin receptor [219]. This chemical modification causes FOXO1 to be sequestered in the cytoplasm, preventing its transcriptional activity [220].

PGC-1α coactivates FOXO1, increasing the expression of gluconeogenic genes such as G6PC and PCK1 in hepatic cells. Insulin counteracts this effect by reducing the mRNAs of these genes without affecting mitochondrial genes controlled by PGC-1α [221]. FOXO1 interacts with the C-terminal domain of PGC-1α (amino acids 551–770), while PGC-1α binds to the N-terminal region of FOXO1. Akt-mediated phosphorylation of FOXO1 disrupts the interaction with PGC-1α, revealing another mechanism by which Akt suppresses FOXO1 activity in addition to nuclear exclusion [221].

FOXO1 transcriptional activity is also regulated by the spliced form of X-box binding protein 1 (XBP1s). XBP1s suppresses FOXO1 by directing it toward proteasomal degradation [222]. PGC-1α interacts with XBP1s, promoting its ubiquitination and degradation, thereby acting as a negative regulator of XBP1s protein levels [223]. This interaction involves amino acids 76–85 within the PGC-1α activation domain, while amino acids 227–252 of XBP1s mediate binding to PGC-1α, with lysines K241 and K257 serving as primary ubiquitination sites [223]. This mechanism ensures that during fasting (when PGC-1α levels rise), the efficiency of the PGC-1α/FOXO1 axis is increased by preventing XBP1s-mediated FOXO1 suppression [223].

#### Ying yang 1 (YY1)

Ying Yang 1 is a widely expressed transcription factor with dual roles, acting as a transcriptional activator and repressor, as its name suggests [224]. It forms homodimers that bind a small sequence motif via four C-terminal zinc fingers [224]. YY1 is essential for central nervous system development [224], cardiac development [225], and for normal skeletal muscle phenotype [226]. Muscle-specific YY1 knockout mice exhibit several skeletal muscle abnormalities, mitochondrial and bioenergetic defects, in a manner that resembles mitochondrial myopathies [226]. Cardiac-specific YY1 knockout mice experience decreased cardiac function in response to exercise, due to blunted adaptive metabolic response caused by downregulation of mRNAs encoding key metabolic genes, including PGC-1α, PPARα, and ERRα [227].

In myotubes, PGC-1α coactivates YY1 to regulate mitochondrial gene expression in an mTOR-dependent manner [228]. mTOR is a serine/threonine kinase that regulates cell growth and metabolism in response to environmental changes [229]. Inhibition of mTOR with rapamycin downregulates mitochondrial genes by preventing the recruitment of PGC-1α to the YY1/mTOR complex bound to gene promoters [228]. The interaction of YY1 with PGC-1α is mediated by the C-terminal region of PGC-1α (amino acids 400–797) and the third C-terminal zinc finger of YY1 (amino acids 353–377) [228]. This interaction depends on mTOR-mediated phosphorylation of YY1 at T30 and S365, as mutation of the phosphorylation sites to alanine residues weakens the binding to PGC-1α and renders YY1 insensitive to the repressive effects of rapamycin [226].

#### Myocyte enhancer factor 2 (MEF2)

The MEF2 family of transcription factors plays a crucial role in regulating muscle-specific gene expression during embryogenesis [230]. In vertebrates, this family consists of four genes—MEF2A, MEF2B, MEF2C, and MEF2D—which exhibit overlapping expression patterns [230].

PGC-1α interacts with MEF2C and acts as its coactivator. In skeletal muscle cells, this interaction promotes the upregulation of GLUT4, the primary insulin-sensitive glucose transporter, whose gene contains a MEF2 binding sequence [231]. While PGC-1α also coactivates MEF2A and MEF2D, its affinity for MEF2C is the strongest [231]. The interaction between PGC-1α and MEF2C specifically involves amino acids 403–570 of PGC-1α and amino acids 93–174 of MEF2C, which correspond to its transactivation domain I [231]. Moreover, MEF2C and MEF2D contribute to the regulation of OCTN2 (organic cation/carnitine transporter 2), a role shared with PPARα. Thus, overexpression of PGC-1α increases OCTN2 mRNA and protein levels in muscle cells [232].

PGC-1α also coactivates MEF2A and MEF2C to induce the expression of carnitine palmitoyltransferase 1β (CPT-1β), an essential mitochondrial membrane protein that transports fatty acids into the mitochondrial matrix for oxidation [233]. However, this function is antagonized by upstream stimulatory factor (USF) proteins, which are widely expressed transcription factors involved in cellular stress responses, differentiation, and proliferation [234]. USF-1/2 directly interacts with the N-terminal transcriptional activation region of PGC-1α (amino acids 1–170) and prevents the PGC-1α-mediated activation of the CPT-1β promoter. In cardiac myocytes, overexpression of USF suppresses PGC-1α-induced CPT-1β expression, returning it to baseline levels [233].

MEF2 factors not only regulate PGC-1α target genes but also control PGC-1α expression itself. The mouse PGC-1α gene contains two MEF2-binding sites, allowing MEF2A, MEF2C, and MEF2D to activate its promoter [235]. However, this effect is counteracted by histone deacetylases (HDACs), which repress transcription. Specifically, HDAC5 can completely inhibit MEF2-mediated PGC-1α promoter activation, acting as a negative regulator [235].

#### p53

p53 is a transcription factor that plays a critical role in cellular responses to extracellular and intracellular stress by regulating genes involved in cell cycle arrest and apoptosis [236–238]. The selection of specific p53 target genes is influenced by post-translational modifications and interactions with transcriptional cofactors and p53-binding proteins [236].

During early glucose starvation (24–36 h), PGC-1α binds to p53 and coactivates it, promoting the expression of genes involved in cell cycle arrest and ROS clearance [239]. However, prolonged starvation (48–72 h) triggers PGC-1α degradation via ubiquitination by the RNF2 E3 ligase, shifting p53 activity toward the induction of pro-apoptotic genes [239]. PGC-1α binding also protects p53 from Lys120 acetylation, a modification known to enhance pro-apoptotic gene expression [240, 241]. Consequently, the loss of PGC-1α facilitates Lys120 acetylation and triggers p53-dependent apoptosis. This interaction occurs through the N-terminal activation domain of PGC-1α (amino acids 1–170) and the transactivation domain of p53 (amino acids 1–43) [239].

Beyond coactivation, p53 also regulates PGC-1α expression in a context-dependent manner. Under metabolic stress or chemically induced glutathione (GSH) depletion, p53 binds to the PGC-1α promoter, increasing PGC-1α expression [242]. However, two additional p53 binding sites within the PGC-1α gene are associated with repression [243]. Upon GSH depletion, occupancy of these repressive sites decreases, while binding to the activating site increases, ultimately leading to increased PGC-1α levels [242]. Notably, despite elevated PGC-1α expression, mitochondrial biogenesis is not induced under these conditions, suggesting that a lower redox state redirects PGC-1α activity toward antioxidant defense rather than energy metabolism [242].

As a tumor suppressor, p53 negatively regulates glycolysis and enhances oxidative phosphorylation [244]. This is particularly relevant in cancer, where cells preferentially rely on glycolysis for energy production, a phenomenon known as the Warburg effect [245]. Certain p53 single nucleotide polymorphisms (SNPs) and mutations, such as P72R, R175H, and R273H, are linked to enhanced metastatic potential [246]. PGC-1α interacts with p53 mutants with differing affinity: in tumor cells harboring the P72 polymorphism and R175H or R273H mutations, mutant p53 binds PGC-1α and inhibits its activity, reducing mitochondrial content and oxygen consumption [247]. In contrast, the R72 polymorphism, alongside R175H or R273H mutations, weakens mutant p53’s interaction with PGC-1α, increasing the pool of available PGC-1α to coactivate other transcription factors, which enables tumor cells to maintain metabolic flexibility—an advantage for metastasis [247]. Consistent with this effect, breast cancer patients with the R72 polymorphism exhibit lower survival rates [247], and high PGC-1α levels in breast cancer are linked to increased metastasis and poor prognosis [248].

The interaction between wild-type p53 and PGC-1α has also been studied in prostate cancer. Overexpression of p53 in PC3 prostate cancer cells suppresses PGC-1α expression, leading to reduced mitochondrial biogenesis and increased apoptosis [249]. These findings suggest a pro-cancer role for PGC-1α in this context, complementing its previously identified function in androgen receptor activation, which may also promote prostate cancer progression [148].

### Integrative analysis of PGC-1α protein-protein interactions

The broad interaction profile of PGC‑1α reflects its role as a central coordinator of metabolic gene expression rather than a conventional transcriptional coactivator with a fixed set of partners. Instead of engaging in a limited number of high‑affinity interactions, PGC‑1α operates through a flexible interaction network that includes transcription factors, chromatin remodelers, coregulators, RNA‑processing proteins, and enzymes responsible for post‑translational modification. Within this network, the functional outcome is determined not simply by the presence of binding partners, but by the specific modes of interaction, domain accessibility, and the regulatory context in which these interactions occur.

A first organizing principle emerges from the topology of PGC‑1α binding. Interactions mediated primarily through the LXXLL (L2) motif conform to the canonical AF‑2–dependent coactivation mechanism characteristic of ligand‑activated nuclear receptors, including PPARs, TRs, RXRs, and LXRs. In contrast, orphan receptors of the ERR family rely predominantly on the L3 (LLKYL) motif, reflecting a mode of regulation that is more directly coupled to PGC-1α abundance due to its constitutive, ligand-independent activity. This distinction helps account for the sustained transcriptional programs driven by ERR–PGC‑1α complexes in high‑energy tissues, compared to the more conditional activation observed for LXXLL‑dependent interactions. A second principle relates to how PGC‑1α recruitment is controlled. In some cases, ligand binding stabilizes receptor conformations that favor coactivator engagement, as observed for PPARs and ERα. In others, recruitment is primarily governed by signaling pathways, including nutrient status, hormonal cues, or cellular stress. Transcription factors such as ERRs, FOXO1, MEF2, YY1, and p53 fall into this latter category. This dual mode of regulation allows PGC‑1α to integrate endocrine signals with intracellular signaling cascades without relying on a single dominant regulatory input.

Not all interactions result in straightforward transcriptional activation. Several partners modify PGC‑1α output through binding competition, altering subnuclear localization, promoting PGC‑1α degradation, or protecting against it. Examples include the competition between PXR, CAR and HNF4α for shared regulatory elements, and CAR-mediated sequestration into nuclear subdomains (PML bodies), followed by PGC-1α ubiquitination and degradation. In parallel, RNA‑associated functions introduce an additional layer of regulation: PGC‑1α can influence the expression of TR isoforms, and in hepatocytes, PGC‑1α stabilizes and promotes export of specific transcripts required for gluconeogenesis while leaving others unaffected. These features indicate that PGC‑1α regulates gene expression through coordinated control of both transcriptional and post‑transcriptional processes. The composition of protein complexes, availability of RNA‑processing machinery, and local signaling environment together determine transcriptional output in a tissue‑specific manner. Although RNA‑mediated functions remain less extensively characterized than canonical coactivation, they provide a mechanism for coupling transcriptional activation with transcript maturation and export.

Within this framework, variability in partner abundance, metabolic state, and proteostasis capacity across tissues produce distinct PGC‑1α‑dependent outputs. The often‑reported pro‑tumor vs. anti‑tumor roles of PGC‑1α can be understood as consequences of partner selection, temporal dynamics, and cellular state. Association with ERRs, AR, or mutant p53 tends to support mitochondrial activity and metastatic potential in metabolically flexible cancer cells. Conversely, interaction with wild‑type p53 or activation under early stress conditions contributes to growth arrest and adaptive responses, with prolonged activation leading to apoptosis. Thus, the oncogenic or tumor-suppressive outcomes attributed to PGC‑1α reflect differences in interaction networks and cellular context rather than intrinsic properties of the coactivator itself.

## Post-translational modifications

**Fig. 5:**
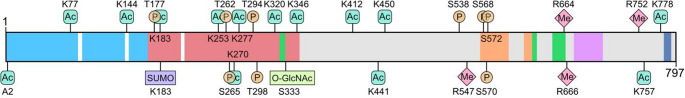
Potential sites for post-translational modifications in murine full-length PGC-1α

PGC-1α is subjected to several chemical modifications that can alter its biological activity by affecting its stability or its binding affinity to transcription factors, other coactivators or corepressors. The post-translational modifications that were identified in the literature are condensed in Table 1 and visually represented in Fig. 5.

**Table 1:** Summary of possible post-translational modifications of PGC-1α, including the name of the enzyme that catalyzes the modification, the PGC-1α substrate amino acids (if known), and potential biological outcome of the modification. Unless specified, sites of modification are for murine PGC-1α

Modification	Enzyme	Amino acids	Biological outcomes	Evidence level	Refs.
Phosphorylation	p38 MAPK	T262, S265, T298	Increased PGC-1α stability and enhanced transcriptional activation via disruption of p160^MBP^ binding	In vitro (enzymatic assays, cell culture)	[250, 251]
Phosphorylation	AMPK	T177, S538	Activation of PGC-1α and enhanced transcription of target genes in skeletal muscle cells	In vitro (enzymatic assays, cell culture) and in vivo (skeletal muscle of wild-type mice and muscle-specific PGC-1α -/- mice)	[252]
Phosphorylation	Akt2/PKB	S570	Decreased binding to chromatin and repression of gluconeogenesis and fatty acid oxidation genes	In vitro (enzymatic assays, cell culture) and in vivo (liver of wild-type mice subject to adenovirus-mediated gene transfer)	[28]
Phosphorylation	Clk2	RS domain	Repression of gluconeogenic genes at later stages of refeeding	In vitro (enzymatic assays, cell culture) and in vivo (liver of wild-type and *db/db* mice, subject to adenovirus-mediated gene transfer)	[253]
Phosphorylation	S6K1	S568, S572	Reduced binding of PGC-1α to HNF4α and repression of gluconeogenesis genes	In vitro (enzymatic assays, cell culture) and in vivo (liver of wild-type mice subject to adenovirus-mediated gene transfer)	[254]
Phosphorylation	GSK3β	T295 (human)	Signaling for PGC-1α ubiquitination by Fbw7	In vitro (enzymatic assays, cell culture) and in vivo (skeletal muscle of calorie-restricted/toxin-exposed wild-type mice)	[255, 256]
Methylation	PRMT1	R665, R667, R669^**a**^ (human)	Enhanced PGC-1α transcriptional activation	In vitro (enzymatic assays, cell culture) and in vivo (adipose tissue of wild-type and PRMT1 adipocyte-specific-–deleted mice)	[257, 258]
Methylation	PRMT1/7	R548, R753 (human)	Putative methylation sites of unknown biological significance	In vitro (enzymatic assays)	[87]
Methylation Demethylation	SET7/9 LSD1	K779 (human)	Increased expression of eRNAs and associated mRNAs	In vitro (cell culture)	[259]
Acetylation	Naa10p	A2	Weakens the interaction between PGC-1α and PPARγ	In vitro (enzymatic assays, cell culture) and in vivo (adipose tissue of Naa10-knockout mice)	[260]
Acetylation Deacetylation	GCN5 SIRT1, HDAC1/3	K77, K144, K183, K253, K270, K277, K320, K346, K412, K441, K450, K757, K778	Acetylation by GNC5 decreases the coactivation ability of PGC-1α, reducing the expression of gluconeogenic genes and mitochondrial genes. Deacetylation mediated by SIRT1 is required to maximize the expression of PGC-1α target genes	In vitro (tagged protein binding, enzymatic assays, cell culture) and in vivo (skeletal muscle/liver of fasted wild-type mice, and skeletal muscle extracted from wild-type mice post-exercise)	[261–265]
O-GlcNAcylation De-GlcNAcylation	OGT O-GlcNAcase	S333	Enhances PGC-1α protein stability and increases hepatic gluconeogenic gene expression	In vitro (cell culture)	[266, 267]
Ubiquitination	Fbw7	-	Conflicting evidence. The cytoplasmatic isoform reduces PGC-1α levels, while the nuclear isoform may have the opposite effect	In vitro (enzymatic assays, cell culture)	[255, 268]
Ubiquitination	RNF34 Cullin1	-	Reduces PGC-1α levels	In vitro (enzymatic assay, cell culture) and in vivo (liver of wild-type and CAR -/- mice)	[168, 269]
Deubiquitination	A20	-	May protect PGC-1α from ubiquitin-targeted degradation	In vivo (adipose tissue of obese humans and obese mice)	[270]
Deubiquitination	BAP1	-	Protects O-GlcNAcylated PGC-1α from degradation	In vitro (cell models)	[267]
SUMOylation	PIAS1/3	K183	Repression of PGC-1α transcriptional activation	In vitro (enzymatic assay, cell models)	[271]
De-SUMOylation	SENP1	K183	Increased PGC-1α transcriptional activation	In vitro (cell models)	[272]

^**a**^ No equivalent residue in murine PGC-1α

### Phosphorylation

Phosphorylation is a common post-translational modification that regulates protein function in response to extracellular signals [273]. This reaction is catalyzed by protein kinases, which can be specific to serine/threonine residues, tyrosine residues, or both (dual-specificity kinases) [274]. Kinases transfer the terminal phosphate ($\:\mathrm{P}{\mathrm{O}}_{3}^{2-}$) group from an ATP molecule to the hydroxyl group of the target amino acid (serine, threonine, or tyrosine), forming a phosphoester bond. This modification is reversible, as protein phosphatases can hydrolyze the bond, removing the phosphate group and restoring the protein to its unphosphorylated state [273].

In the case of PGC-1α, phosphorylation catalyzed by p38 mitogen-activated protein kinase (MAPK) can occur at three sites (T262, S265, T298), all located within the negative regulatory domain of PGC-1α [250]. As a consequence, PGC-1α protein stability is enhanced, as evidenced by a 2.5-fold increase in half-life relative to the unphosphorylated protein [250]. This modification can be triggered by inflammatory cytokines and β₃-adrenergic signaling, and also promotes mitochondrial biogenesis, as reflected by increased expression of genes such as cytochrome c, COX2, COX4, and ATP synthase subunit β, leading to elevated oxygen consumption [250]. The mechanism underlying the enhanced stability of the triple-phosphorylated PGC-1α remains unclear, but may involve altered interactions with components of proteolysis pathways [250].

Beyond the stabilization effect, phosphorylation by p38 MAPK disrupts the association of PGC-1α with p160 myb-binding protein (p160^MBP^), a transcriptional repressor that binds to the PGC-1α negative regulatory domain (200–400) and suppresses its activity [251]. Thus, this mechanism explains, at least in part, the increased transcriptional activity of phosphorylated PGC-1α [250]. The interaction between PGC-1α and p160^MBP^ relies on the L2 and L3 leucine-rich motifs of PGC-1α, as mutations in these sequences abolish binding and prevent the p160^MBP^-mediated repression [251]. The repressive function of p160^MBP^ appears to involve the recruitment of histone deacetylases, as the addition of a histone deacetylase inhibitor attenuates its effects [251].

PGC-1α is also targeted by other protein kinases. In skeletal muscle, the AMP-activated kinase (AMPK) phosphorylates PGC-1α at T177 and S538, resulting in PGC-1α activation and enhanced transcription of genes involved in glucose uptake (GLUT4) and mitochondrial function (cytochrome c and PGC-1α itself). These outcomes suggest that phosphorylation of these sites modulate the ability of PGC-1α to interact with certain transcription factors or other coactivators/corepressors [252].

Conversely, phosphorylation at S570 by Akt2/PKB in the liver exerts an inhibitory effect, suppressing the transcription of gluconeogenic and fatty acid oxidation genes. This modification occurs in response to insulin signaling and reduces PGC-1α occupancy of promoter regions, leading to decreased expression of PCK1, G6PC, and MCAD [28]. Similarly, Cdc2-like kinase 2 (Clk2) phosphorylates the RS domain of PGC-1α during late refeeding phases, suppressing many PGC-1α target genes, but with a more potent inhibitory effect on gluconeogenic genes [253]. A more selective inhibition of gluconeogenic genes is evident with the serine/threonine kinase S6K1 (S6 kinase 1), which also phosphorylates residues within the RS domain (S568 and S572), resulting in impaired binding of PGC-1α to HNF4α, without affecting its interactions with other nuclear receptors such as PPARα and ERRα [254]. Consequently, while mitochondrial and fatty acid oxidation pathways remain largely unaffected, gluconeogenic gene expression is selectively downregulated [254].

An inhibitory effect on PGC‑1α activity mediated by phosphorylation has also been described for glycogen synthase kinase 3β (GSK3β) [255, 256]. GSK3β is a ubiquitously expressed serine/threonine kinase that is constitutively active, and preferentially phosphorylates substrates that have been pre‑phosphorylated by a priming kinase at a serine or threonine residue located at position +4 (C-terminal) within the consensus sequence (S/T)XXX(S/T) [275]. Phosphorylation by GSK3β frequently serves as a signal for subsequent ubiquitination and proteasomal degradation [276]. In the context of PGC‑1α regulation, Anderson et al. [256] provided initial evidence that GSK3β phosphorylation contributes to reduced PGC‑1α protein levels. In NIH3T3 mouse embryonic fibroblasts, siRNA‑mediated depletion of GSK3β was associated with increased nuclear immunofluorescent staining of PGC‑1α, consistent with enhanced stability. Subsequent work by Olson et al. [255] established a mechanistic connection between GSK3β phosphorylation and ubiquitin‑mediated degradation of PGC‑1α through the E3 ligase Fbw7 (F-box and WD repeat domain-containing 7), discussed in detail in the “Ubiquitination” subsection. Two candidate Fbw7-binding motifs, also called Cdc4 phosphodegrons (CPD), were identified in PGC‑1α (CPD1 and CPD2), with site‑directed mutagenesis indicating that CPD2 functions as the dominant degron. This region contains T299 (T298 in mouse PGC‑1α), which overlaps with a previously identified p38 MAPK phosphorylation site. p38 MAPK–dependent phosphorylation at this position therefore acts as a priming event that enables subsequent GSK3β phosphorylation at T295, facilitating Fbw7 recognition. Because p38 MAPK phosphorylation also enhances PGC‑1α transcriptional activity, this arrangement couples transcriptional activation and degradation to the same signaling cascade, ensuring that PGC‑1α activation remains transient [255].

In addition to modulating transcriptional activity and protein stability, phosphorylation has been proposed to influence the subcellular localization of PGC‑1α. In resting skeletal muscle, PGC‑1α is predominantly cytosolic, and acute endurance exercise promotes nuclear accumulation in both rodents and humans [19, 277]. In trained individuals, acute endurance exercise resulted in a > 50% increase in nuclear PGC‑1α content without detectable changes in total cellular PGC‑1α protein levels, coinciding with robust activation of AMPK immediately post‑exercise [277]. These observations suggest that exercise‑activated kinases such as p38 MAPK and AMPK may promote nuclear accumulation of PGC‑1α independently of changes in expression or stability. Despite correlative evidence, the molecular mechanisms governing phosphorylation-mediated PGC‑1α nuclear translocation remain unresolved. Phosphorylation can influence nucleocytoplasmic trafficking through multiple mechanisms, including direct masking or unmasking of NLSs or nuclear export signals (NES), or regulation of interactions with binding partners that control nuclear import or export [278]. However, there is no concrete evidence that p38 MAPK‑ or AMPK‑dependent phosphorylation alters PGC‑1α nuclear trafficking through any of these mechanisms. In contrast, the subcellular trafficking of the truncated isoform NT‑PGC‑1α has been mechanistically characterized in greater detail. NT‑PGC‑1α lacks canonical NLS motifs and is predominantly cytosolic, with nuclear export mediated by the exportin CRM1 (chromosome region maintenance 1) through two identified NES motifs (amino acids 27–36 and 90–99) [72]. Phosphorylation of NT‑PGC‑1α by protein kinase A (PKA) at Ser194, Ser241, and Thr256 inhibits CRM1‑dependent nuclear export, resulting in nuclear accumulation [72]. Although NT‑PGC‑1α shares substantial sequence identity with full‑length PGC‑1α—including putative NES elements—inhibition of CRM1 does not alter the subcellular localization of the canonical PGC‑1α isoform [72], indicating the presence of distinct or additional regulatory mechanisms governing the trafficking of the full-length protein.

### Methylation

Arginine methylation is catalyzed by protein arginine methyltransferases (PRMTs), which transfer a methyl group from S-adenosylmethionine to the nitrogen atoms in arginine’s guanidine group [279]. This modification can occur in three forms: monomethylation, symmetric dimethylation (one methyl group attached to each guanidino nitrogen), or asymmetric dimethylation (two methyl groups attached to a single guanidino nitrogen) [279, 280]. In mammals, there are nine PRMT members, classified into three types based on their catalytic activity: type I (PRMT1–4, PRMT6, PRMT8), type II (PRMT5, PRMT9), and type III (PRMT7) [279]. In general, arginine methylation plays diverse roles in gene regulation, including modulation of transcription factors and coactivators/corepressors, as well as direct regulation of transcription through histone methylation [280].

Teyssier et al. first identified PRMT1 as the enzyme responsible for methylating arginine residues (R665, R667, R669) in the C-terminal acidic glutamate-rich region of PGC-1α [257]. They found that this modification enhanced the coactivation function of PGC-1α, since a methylation-deficient PGC-1α mutant (arginines replaced with lysines) showed reduced efficiency in inducing ERRα and cytochrome c expression. Consistently, siRNA-mediated PRMT1 knockdown partially impaired wild-type PGC-1α ability to activate these genes. Based on this, it is likely arginine methylations promote structural changes in PGC-1α, improving interactions with coactivators or reducing affinity towards corepressors [257].

Additionally, PRMT1 splicing variants, particularly PRMT1V2, participate in cold-induced thermogenesis by coactivating PGC-1α in adipocytes [258]. In adipocyte-specific PRMT1 knockout mice, thermogenic and adipogenic gene expression remained unchanged at room temperature (23 °C). However, under cold stress (10 °C), these knockout mice exhibited impaired ability to regulate body temperature, with reduced expression of thermogenic, mitochondrial, and fatty acid oxidation genes. When PRMT1V2 was coexpressed with PGC-1α in cultured PRMT1 -/- adipocytes, PGC-1α strongly induced thermogenic gene expression, while coexpression of PRMT1V1 had a minimal effect, highlighting the dominant role of the PRMT1V2 variant in thermogenic regulation [258].

PGC-1α may also be methylated by PRMT7 [87], a type III PRMT which preferentially targets arginine residues within RXR motifs [281] and regulates PGC-1α expression in skeletal muscle [282]. In vitro methylation assays detected monomethylation of PGC-1α C-terminal fragments at R548 and R753 by both PRMT7 and PRMT1, with additional potential methylation sites predicted computationally but not confirmed experimentally [87]. However, the biological relevance of these newly detected methylation sites remains unclear due to the in vitro nature of the study.

PGC-1α can also be subject to lysine methylation catalyzed by the histone mono-methyltransferase SET7/9, and reversed by lysine-specific demethylase 1 (LSD1) [259]. Methylation at K779 enhances PGC-1α-induced expression of certain genes, by facilitating interactions with the SAGA (Spt-Ada-Gcn5 acetyltransferase) and Mediator complexes. Recruitment of these complexes at enhancer regions leads to increased expression of eRNAs (enhancer RNAs), which strongly correlate to mRNA levels of nearby genes. The stability of eRNAs is augmented by RNA methylation, in particular cytosine to 5-methylcytosine, catalyzed by the NSUN7 (NOP2/Sun RNA methyltransferase family member 7). NSUN7 can interact with both methylated and unmethylated PGC-1α and may aid in fine-tuning the regulation of gene expression in response to metabolic state. NSUN7 levels rise during fasting, which leads to increased methylated RNA and eRNAs, enhancing the expression of specific PGC-1α target genes [259].

### Acetylation

Lysine acetylation is a modification catalyzed by lysine acetyltransferases, which transfer an acetyl group (COCH_3_) from acetyl-CoA to the ε-amino group of lysine residues. Lysine acetyltransferases can be grouped into different families according to sequence similarity, with the main groups GCN5/PCAF, p300/CBP, and MYST [283]. This modification can be reversed by lysine deacetylases, which catalyze the hydrolysis of ε-acetyl-lysine residues [284]. Lysine deacetylases are grouped in two large families according to the reaction mechanism: Zn^2+^-dependent histone deacetylases (HDACs) and sirtuins (SIRTs), which are NAD^+^-dependent lysine deacetylases [285, 286]. Lysine acetylation is involved in diverse cellular processes, including chromatin remodeling, gene transcription, and metabolic regulation, by altering protein-DNA and protein-protein interactions, subcellular localization, and enzymatic activity [287].

For PGC-1α specifically, lysine acetylation is tightly linked to the cell’s adaptive response to fluctuations in nutrient status [288]. This regulation is mediated by changes in the concentrations of key metabolic cofactors: (i) acetyl-CoA, produced during glucose or fatty acid oxidation, which serves as the acetyl donor for acetyltransferases, and (ii) NAD⁺, whose levels rise relative to NADH during nutrient-deprived states, and acts as a cosubstrate for sirtuin deacetylases. Consequently, shifts in nutrient availability are directly transduced into changes in acetylation state, coupling metabolic signals to transcriptional programs that govern energy homeostasis [288].

PGC-1α can undergo acetylation at 13 lysine sites (amino acids 77, 144, 183, 253, 270, 277, 320, 346, 412, 441, 450, 757, 778), which was demonstrated to diminish its ability to coactivate HNF4α [262]. The acetylation reactions are catalyzed by GCN5 (general control non-depressible 5), which interacts with the N-terminal region of PGC-1α, and broadly acts to repress the transcriptional activation mediated by PGC-1α on gluconeogenic and mitochondrial genes alike [263]. Coexpression of GCN5 and PGC-1α in hepatocytes revealed that acetylation redistributes PGC-1α to inactive nuclear domains where it colocalizes with nuclear corepressors such as RIP140 (receptor interacting protein 140 kDa). The relocalization of PGC-1α is dependent on its C-terminal region, as truncated variants (amino acids 1–570) did not differ from the wild-type protein with regards to nuclear distribution [263].

The reverse reaction is catalyzed by SIRT1, an enzyme that physically interacts with and deacetylates PGC-1α in a NAD^+^-dependent manner [261]. In hepatocytes, SIRT1 inhibition via nicotinamide treatment resulted in a 24-fold reduction of PGC-1α/HNF4α transcription activity. Consistent with this, SIRT1 knockdown blocked PGC-1α-induced transcription of gluconeogenic genes (PCK1, G6PC), with little effect on mitochondrial genes (cytochrome c, β-ATP synthase) in these cells [262]. In skeletal muscle cells, nutrient deprivation states (i.e., increased NAD^+^/NADH) enhance SIRT1 deacetylase activity, reversing PGC-1α acetylation and resulting in upregulation of mitochondrial and fatty acid oxidation genes [264]. Additionally, SIRT1 knockdown via shRNA significantly reduced expression of these genes, as well as mitochondrial regulators (ERRα, TFAM), highlighting its role in maximizing PGC-1α-driven transcription [264]. Thus, SIRT1 acts as a nutrient sensor, responding to increases in NAD^+^ levels by deacetylating PGC-1α and enhancing the transcription of gluconeogenesis genes in hepatocytes [262] and fatty acid oxidation genes in skeletal muscle to spare glucose for other tissues [264, 289].

As highlighted in the **“**Phosphorylation” subsection, AMPK modulates PGC-1α activity directly via phosphorylation [252], but it is also able to act indirectly, by enhancing SIRT1-mediated deacetylation [265]. In response to a rising AMP/ATP ratio, AMPK enhances fatty acid β-oxidation metabolism, which elevates the cellular NAD⁺/NADH ratio and activates SIRT1. Treatment of cultured skeletal muscle cells with etomoxir, a mitochondrial fatty acid β-oxidation inhibitor, abolished AMPK-induced PGC-1α deacetylation. Consistently, a PGC-1α mutant lacking AMPK phosphorylation sites showed reduced deacetylation and failed to upregulate mitochondrial genes in the presence of the AMPK activator AICAR (5-amino-4-imidazolecarboxyamide ribonucleoside). These findings suggest that phosphorylation serves as a priming signal for SIRT1-mediated deacetylation, ensuring SIRT1 selectively deacetylates relevant substrates [265].

In addition to enhancing transcriptional coactivation, SIRT1‑mediated deacetylation of PGC‑1α has been proposed to influence its subcellular distribution [256]. In NIH3T3 mouse embryonic fibroblasts under basal conditions, PGC‑1α is detected in both cytoplasmic and nuclear compartments. Upon exposure to oxidative stress induced by hydrogen peroxide, PGC‑1α accumulated in the nucleus, an effect attenuated by the SIRT1 inhibitor nicotinamide, consistent with a role for deacetylation in facilitating nuclear accumulation. This redistribution was transient, with nuclear PGC‑1α levels returning to baseline one hour after stimulation. The decline in nuclear protein abundance was associated with ubiquitin‑mediated degradation and required prior phosphorylation of PGC‑1α by GSK3β [256]. These findings support a model in which the duration of PGC‑1α–dependent transcriptional responses to oxidative stress are constrained by intranuclear turnover.

While SIRT1-mediated deacetylation of PGC-1α has been extensively characterized, recent studies have explored the ability of histone deacetylases (HDACs) to target PGC-1α [290, 291]. HDAC3 is a critical regulator of PGC-1α activity in BAT, where it deacetylates PGC-1α and enables its coactivation of ERRα to sustain basal thermogenic capacity [290]. In BAT-specific HDAC3 knockout mice, mitochondrial oxidative phosphorylation genes were profoundly downregulated, accompanied by diminished expression of UCP1 and impaired function of electron transport chain complexes. Mechanistically, HDAC3 reverses GCN5-mediated acetylation of PGC-1α both in vivo and in vitro, thereby restoring its transcriptional activation ability over ERRα bound to enhancer regions of target genes. This deacetylation event is essential for ERR-driven transcription of UCP1, oxidative phosphorylation genes, and PPARGC1A itself. Because PGC-1α participates in a positive feedback loop by promoting transcription of its own gene, HDAC3 deficiency also reduces PGC-1α mRNA and protein levels in BAT, amplifying the thermogenic defect [290].

Similarly, HDAC1 has emerged as an important regulator of hepatic gluconeogenesis [291]. In hepatocytes, HDAC1 deacetylates PGC-1α and enhances its ability to coactivate HNF4α, promoting the transcription of gluconeogenic genes. Knockdown of HDAC1 suppresses glucagon-stimulated glucose production and reduces the expression of G6PC, even under conditions of ectopic PGC-1α overexpression, whereas PCK1 expression remains largely unaffected, suggesting the presence of additional regulatory inputs. Liver-specific depletion of HDAC1 in diet-induced obese mice lowers fasting blood glucose, although gluconeogenic gene expression is not reduced, indicating the presence of compensatory mechanisms in vivo [291].

In addition to acetylation of internal lysine residues, the N-terminus of PGC-1α can be acetylated by Naa10p, an N-α-acetyltransferase (NAT) that catalyzes the N-α-acetylation of nascent proteins [260]. Unlike lysine acetylation, this modification is typically irreversible [283], and it may be preceded by cleavage of the N-terminal methionine residue by methionine aminopeptidases [292]. Depending on the NAT type, N-α-acetylation can occur in parallel to mRNA translation (co-translationally), once the nascent protein is sufficiently large, or post-translationally [292, 293]. Depending on the substrate, N-α-acetylation may affect protein folding, protein-protein interactions, subcellular localization, or protein stability [293].

This modification was shown to disrupt the interaction of PGC-1α with PPARγ, repressing beige adipocyte marker genes such as UCP1 [260, 294]. Naa10p knockout (KO) mice exhibited resistance to high-fat diet-induced obesity, showing 20–50-fold higher UCP1 expression, increased oxygen consumption, and greater energy expenditure. This effect was specifically linked to Naa10p’s acetylase activity, as a mutant lacking enzymatic activity (R82A) failed to repress UCP1 in cultured beige adipocytes. Co-immunoprecipitation revealed that Naa10p KO increased PGC-1α binding to PPARγ, an effect mimicked by the R82A mutant but not the wild-type protein. Similarly, a PGC-1α mutant resistant to N-α-acetylation, where the N-terminal Ala residue (after excision of Met1) is replaced by Pro, replicated the thermogenic gene expression and PPARγ interaction seen in Naa10p KO models [260].

### O-GlcNAcylation

O-GlcNAcylation is a glucose-derived post-translational modification that regulates transcriptional and translational processes, in a manner analogous to phosphorylation [295]. The hexosamine biosynthetic pathway converts glucose into UDP-GlcNAc (uridine diphosphate-β-N-acetylglucosamine), which serves as the donor substrate for OGT (O-linked N-acetylglucosaminyltransferase), the enzyme that catalyzes O-GlcNAcylation of serine and threonine residues. This modification is reversible, with O-GlcNAcase mediating its removal [295].

Elevated UDP-GlcNAc levels are linked to insulin resistance [296, 297] and enhance O-GlcNAcylation of FOXO transcription factors in the liver, promoting the expression of gluconeogenesis enzymes [298]. However, O-GlcNAcylation also plays a protective role in cellular stress responses, as glucose uptake increases independently of energy status during several stress conditions [295]. For example, O-GlcNAcylation increases the expression of heat shock proteins in response to thermal stress [299] and promotes antioxidant defense by increasing the expression of ROS detoxifying enzymes such as catalase via FOXO1 [298]. Additionally, O-GlcNAcylation is essential for embryonic development, as severe disruption of OGT activity is lethal [300].

Housley et al., employing mass spectrometry analysis, identified that PGC-1α can be O-GlcNAcylated at Ser333 [266]. Later research showed that this modification has a stabilizing effect by enhancing the binding of BAP1 (BRCA1-associated protein 1), a deubiquitinase that prevents PGC-1α degradation via the ubiquitin-proteasome pathway [267]. The recruitment of OGT to catalyze the O-GlcNAcylation of PGC-1α is mediated by HCF-1 [267]. Consequently, the PGC-1α mutant Y385A, which is unable to bind HCF-1, cannot be O-GlcNAcylated, and exhibits increased ubiquitination compared to the wild-type protein. In a similar fashion, mutation of the known O-GlcNAcylation site (S333A), also leads to increased susceptibility to ubiquitination [267]. Because of the increased PGC-1α levels due to O-GlcNAcylation, gluconeogenesis is promoted by enhancing FOXO1 and HNF4α-mediated transcription of gluconeogenic genes such as G6PC and PCK1 [267]. The O-GlcNAcylation of PGC-1α depends on cellular glucose levels and peaks under euglycemic conditions, indicating that OGT/HCF-1/PGC-1α has a role in maintaining glucose homeostasis [267].

In addition to being a substrate for O-GlcNAcylation by OGT, PGC-1α recruits transcription factors, such as FOXO1 and FOXO3, for OGT-mediated O-GlcNAcylation [266]. As a result of O-GlcNAcylation, FOXO1 and FOXO3 exhibit increased transcriptional activation. Consistent with this, overexpression of PGC-1α in Fao rat hepatoma cells amplifies O-GlcNAcylation of FOXO1 [266]. The physiological relevance of this mechanism remains elusive, as O-GlcNAcylation increases with glucose intake, while PGC-1α is typically upregulated during fasting and activates FOXO1 to promote gluconeogenic gene expression. Nevertheless, in diabetes, excessive O-GlcNAcylation of FOXO1 may contribute to the dysregulated gluconeogenesis state evident in this metabolic disorder [266]. Furthermore, in the liver of diabetic mice, elevated BAP1 expression contributes to persistent gluconeogenesis, such that OGT/HCF-1 knockdown leads to normalization of blood glucose levels, mitigating diabetes-related metabolic dysfunction [267].

The investigation of a naturally occurring SNP of pig PGC-1α, where cysteine at position 430 is replaced by serine (C430S), revealed altered transcriptional activity associated with increased O-GlcNAcylation [301]. The C430S variant, which is more susceptible to O-GlcNAcylation, exhibits enhanced stability relative to the wild-type protein, corroborating with previous research [267]. However, the C430S mutant paradoxically induces the transcription of PCK1 to a significantly lesser extent than wild-type PGC-1α. This modification appears to weaken the interaction between PGC-1α and PPARγ, leading to the observed reduced transcriptional activation of PCK1, a gene under regulatory control of PPARγ/PGC-1α [301].

While O-GlcNAcylation results in increased PGC-1α levels under physiological conditions, it has been associated with a decline of PGC-1α mRNA and protein levels under cardiac hypertrophy [302]. In this pathological state, overexpression of O-GlcNAcase reverts the observed decline of PGC-1α mRNA levels. In glucose-starved cardiac cells, where PGC-1α levels are typically elevated, treatment with glucosamine or PUGNAc (an O-GlcNAcase inhibitor) reduces PGC-1α expression and suppresses its target fatty acid metabolism and mitochondrial genes. Consistent with this, OGT knockout cells exhibit increased PGC-1α/β mRNA levels as well as increased expression of PGC-1α target genes. Thus, O-GlcNAcylation signaling appears to contribute to the reduction of PGC-1α levels during cardiac hypertrophy, although it is insufficient to induce this pathological state. Despite the positive effect of complete OGT depletion on PGC-1α expression, lack of OGT eventually leads to progressive cardiomyopathy, underscoring its essential role in the maintenance of cardiomyocyte physiology [302].

### Ubiquitination

Ubiquitination is a three-step enzymatic cascade that covalently attaches ubiquitin, a highly conserved polypeptide, to a target protein [303]. The process begins with ubiquitin “activation” by an E1 ubiquitin-activating enzyme, forming a high-energy thiol ester bond between the C-terminal glycine of ubiquitin and a cysteine residue in E1 [303]. Next, ubiquitin is transferred to an E2 ubiquitin-conjugating enzyme through a transthiolation reaction at the E2 active site cysteine [304]. In the final step, the ubiquitin-loaded E2 interacts with an E3 ubiquitin ligase, which facilitates the transfer of ubiquitin to the substrate. This transfer can occur directly from E2 to the substrate or via formation of an E3-ubiquitin intermediate [303]. In the direct pathway, E3 serves as a scaffold, positioning E2 for efficient ubiquitin transfer, whereas in the alternative pathway, ubiquitin is transferred from E2 to E3 through an additional transthiolation reaction before being covalently attached to the substrate [305].

Typically, ubiquitin is conjugated to an exposed ɛ-NH_2_ group of a lysine residue within the substrate, although it can also be attached to the N-terminal α-amino group [303]. Once a ubiquitin molecule is attached, additional ubiquitin units can be linked to one of its seven internal lysines or to its N-terminal amino group, forming polyubiquitin chains with distinct structural configurations. These chains serve as molecular signals, directing proteins toward degradation or regulating non-proteolytic cellular processes [306]. In the case of proteolytic signaling, polyubiquitinated proteins are recognized by the 26S proteasome, a large macromolecular complex responsible for targeted protein degradation. The 26S proteasome consists of two subcomplexes: the 20S core particle, responsible for cleaving proteins into peptides, and the 19S regulatory particle, which recognizes ubiquitinated substrates and facilitates their translocation into the core for degradation [307].

PGC-1α protein levels are regulated by degradation via the ubiquitin-proteasome pathway, with multiple E3 ligases implicated in this process. Olson et al. [255] demonstrated that the ubiquitin ligase complex SCF^Fbw7^—where the component Fbw7 mediates substrate recognition—targets PGC-1α for degradation. In humans, the Fbw7 gene encodes three isoforms generated by alternative splicing, each with different subcellular localizations: Fbw7α (the most abundantly expressed, nuclear), Fbw7β (cytoplasmic), and Fbw7γ (nucleolar) [308]. Substrate recognition by Fbw7 depends on the presence of a Fbw7-binding consensus motif (CPD), typically following the pattern (T/S)PPX(T/S), albeit some sequence variability is tolerated [309]. This motif contains two phosphorylatable residues at position 0 and 4, where phosphorylation at position 0 is essential for Fbw7 binding, while phosphorylation at position 4 enhances binding affinity [310]. Importantly, the Fbw7 CPD overlaps directly with the consensus sequence targeted by GSK3β, which generates the dual-phosphorylation pattern on primed (pre-phosphorylated) substrates for optimal Fbw7 binding [311]. Among the two sequences conforming to CPDs on PGC-1α (CPD1 and CPD2) identified by Olson et al. [255], mutation of phosphorylation sites in CPD2 alone was found to significantly reduce binding of PGC-1α to Fbw7γ, indicating CPD2 is the primary degron. Reporter assays using luciferase constructs showed that expression of nuclear Fbw7 isoforms (Fbw7α or Fbw7γ) led to markedly decreased PGC-1α-driven transcription, whereas only a minor reduction in transcriptional activity was observed with the cytoplasmic isoform (Fbw7β). These findings position GSK3β‑dependent phosphodegron formation as a critical regulatory step linking prior activating phosphorylation events to Fbw7‑mediated degradation of PGC‑1α within the nucleus [255].

Additional evidence supporting a role for Fbw7 in PGC‑1α turnover was provided by Park et al. [312], who examined the function of Ewing sarcoma protein (EWS), a transcription factor involved in brown adipocyte differentiation. In EWS‑deficient brown preadipocytes, PGC‑1α protein levels were reduced and ubiquitination increased, coincident with elevated expression of Fbw7 [312]. Depletion of Fbw7 in these cells with siRNA restored PGC‑1α abundance and increased mitochondrial content, consistent with Fbw7‑dependent degradation of PGC‑1α. Although EWS was found to associate weakly with both endogenous and FLAG‑tagged PGC‑1α, the interaction was not sufficient to establish a direct mechanistic role in regulating PGC‑1α stability [312]. In vivo, EWS knockout mice exhibited reduced mitochondrial abundance in skeletal muscle and decreased expression of mitochondrial biogenesis, respiratory, and fatty acid oxidation genes in the liver, while gluconeogenic gene expression remained largely unaffected [312]. However, given the modest biochemical interaction between EWS and PGC‑1α, these systemic metabolic phenotypes cannot be attributed exclusively to altered PGC‑1α stability and may also reflect EWS interactions with other transcriptional regulators or coactivators. Moreover, the molecular mechanism by which EWS modulates Fbw7 abundance or activity, and how this axis interfaces with established PGC‑1α degradation pathways, remains to be defined.

Trausch-Azar et al. [268] reported findings that contrast with Olson et al. [255], presenting evidence that Fbw7β, rather than Fbw7α, promotes PGC-1α degradation. They found that Fbw7β accelerated ubiquitin-proteasome–dependent degradation by generating high molecular weight ubiquitin conjugates, whereas Fbw7α produced low molecular weight conjugates that do not efficiently signal degradation and instead stabilize PGC-1α. In Fbw7 knockout cells, expression of Fbw7β only slightly reduced PGC-1α half-life (from $\:{t}_{1/2}=$ 0.6 h to $\:{t}_{1/2}=$ 0.5 h), indicating Fbw7 is not the primary E3 ligase for PGC-1α. Moreover, the lack of colocalization between Fbw7β and PGC-1α supports the hypothesis that Fbw7β might exert its effects indirectly, possibly by targeting other proteins that influence PGC-1α stability [268]. The authors propose that discrepancies with Olson et al. [255] may have resulted from the use of N-terminally FLAG-tagged PGC-1α, which can interfere with degradation dynamics. When FLAG-tagged PGC-1α was tested, both Fbw7α and Fbw7β reduced FLAG-PGC-1α levels [268].

Trausch-Azar et al. [95] also provided evidence that nuclear degradation of PGC-1α occurs primarily via an N-terminal ubiquitination subpathway. Their experiments demonstrated that a truncated nuclear construct of PGC-1α (amino acids 1–182) is rapidly degraded, while a longer cytoplasmic isoform (NT-PGC-1α, amino acids 1–270) remains stable. Mutating all lysines in the nuclear construct did not alter degradation rates, suggesting a lysine-independent mechanism. Conversely, Sano et al. [313] proposed that the C-terminal region plays a dominant role in PGC-1α degradation. They found that a FLAG-tagged C-terminal deletion mutant (amino acids 1–565) was resistant to ubiquitination, whereas an N-terminal truncated variant (amino acids 292–798) mimicked proteasome inhibition by forming nuclear aggregates [313].

The divergent conclusions drawn from these studies may once again be caused by the use of epitope or fluorescently tagged PGC‑1α constructs, which can affect protein localization, functionality, and stability. Consistent with this possibility, Anderson et al. [256] reported that overexpressed GFP‑tagged PGC‑1α exhibits a nuclear distribution distinct from that of the endogenous protein. More generally, a systematic assessment by Weill et al. [314] demonstrated that fluorescent tagging alters the cellular distribution of over 500 yeast proteins in a manner dependent on tag position (N or C terminus). Recent studies have further shown that different tagging strategies can affect not only protein localization but also functional properties and interaction networks in biological condensates [315, 316]. These observations indicate that experiments relying on tagged PGC‑1α constructs may introduce variables that complicate interpretation of degradation kinetics and subcellular compartmentalization, potentially contributing to inconsistencies reported for Fbw7‑mediated regulation.

Recent work by Eleuteri et al. [317] expanded the role of Fbw7 in PGC-1α regulation by linking it to chaperone-mediated autophagy (CMA). CMA is a lysosomal degradation pathway that targets cytosolic proteins bearing a specific peptide recognition motif (KFEQR-like motif); the process involves recognition of substrates bearing this motif by the constitutively expressed chaperone protein heat shock cognate 71 kDa (HSC70), which then directs them to lysosome-associated membrane protein type 2A (LAMP-2A), a transmembrane protein found in the lysosomal membrane [318]. This is followed by translocation of the substrate into the lysosome lumen, and subsequent degradation by lysosomal proteases [319]. According to the mechanism proposed by Eleuteri et al. [317], CMA regulates Fbw7β levels, which indirectly affects PGC-1α protein levels, as suggested by pharmacological activation of CMA resulting in reduced Fbw7 protein levels (~ 0.4–0.6-fold) and increased PGC-1α levels (~ 3-fold). In contrast with prior findings by Trausch-Azar et al. [268], the authors report substantial colocalization (~ 40%) between Fbw7β and PGC-1α and show that Fbw7 knockdown or knockout significantly increases PGC-1α protein levels (~ 2–2.5 fold) [317], suggesting a stronger contribution of Fbw7β to PGC-1α turnover than previously presented by Trausch-Azar et al. [268]. These conflicting results may be due to methodological differences between the two studies (e.g., measurements of steady-state protein levels vs. half-life measurements), cell-type specificities (SH-SY5Y vs. HeLa) which could affect PGC-1α subcellular localization (as seen in skeletal muscle, where PGC-1α is primarily cytoplasmic under basal conditions), or the presence of additional indirect effects mediated by CMA modulation.

In another recent study, Zhuang et al. [320] provided complementary but conceptually distinct evidence for the role of CMA autophagy in regulating PGC‑1α protein levels. In contrast to the indirect mechanism proposed by Eleuteri et al. [317], whereby CMA activation reduces Fbw7β abundance and thereby stabilizes PGC‑1α, Zhuang et al. [320] demonstrated that PGC‑1α itself can act as a direct CMA substrate under conditions of thermal stress. In vitro experiments revealed that at elevated temperatures (39 °C), pharmacological inhibition of proteasome degradation could not fully suppress PGC‑1α degradation, which was only possible by concomitant administration of an autophagy inhibitor, indicating a shift toward lysosome‑dependent turnover. The authors identified three conserved KFERQ‑like motifs within the PGC‑1α sequence and showed that mutation of these motifs abolished PGC‑1α lysosomal degradation. Consistent with the presence of a CMA-dependent PGC‑1α degradation mechanism, in vivo knockdown of the CMA receptor LAMP‑2A led to increased PGC‑1α levels and UCP1 expression in brown adipose tissue at thermoneutrality. Furthermore, Zhuang et al. [320] identified PARK7 (Parkinson disease protein 7) as a modulator of CMA flux that competes with HSC70 for interaction with LAMP‑2A and acts as a temperature‑sensitive inhibitor of PGC‑1α degradation; this inhibitory effect was relieved under thermal stress, permitting CMA‑dependent clearance of thermogenic proteins. Taken together, these findings suggest that CMA may exert dual and context‑dependent control over PGC‑1α levels: indirectly stabilizing PGC‑1α through regulation of Fbw7β in certain cellular settings, while directly promoting PGC‑1α degradation in others, depending on cell type, subcellular localization, and environmental conditions such as thermal stress. Whether these mechanisms represent distinct regulatory modes or reflect methodological and contextual differences between studies remains to be determined.

Beyond Fbw7, other E3 ligases have been implicated in PGC-1α regulation. Wei et al. [269] identified RNF34 (ring finger protein 34) as a nuclear E3 ligase that targets PGC-1α for degradation. Coexpression of hemagglutinin-tagged PGC-1α with RNF34 led to a near-complete reduction in PGC-1α levels, an effect reversed by proteasome inhibition. The C-terminal half of PGC-1α is required for RNF34 binding, as RNF34 selectively reduces the levels of a truncated C-terminal construct (amino acids 350–797) but has no effect on N-terminal constructs. In rat hepatoma cells, RNF34 overexpression abolished PGC-1α-induced activation of gluconeogenic (PDK4) and fatty acid oxidation genes (MCAD) while leaving unaffected genes that do not rely on PGC-1α, such as UCP2. Similarly, in brown adipocytes, RNF34 overexpression reduced endogenous PGC-1α protein levels without affecting its mRNA, leading to decreased expression of PGC-1α target genes (e.g., UCP1) and reduced oxygen consumption [269].

More recently, Zhao et al. [321] demonstrated that lysine methyltransferase 5 (KMT5C) competes with RNF34 for binding to the C-terminal region of PGC-1α (amino acids 565–797), thereby reducing RNF34 association and limiting PGC-1α ubiquitination and proteasomal degradation. This occurs independently of KMT5C’s methyltransferase activity, as a catalytically inactive mutant reproduces the stabilizing effect on PGC-1α. Overexpression of KMT5C in hepatocytes selectively upregulates gluconeogenic genes such as PCK1 and G6PC through HNF4α coactivation, without affecting fatty acid oxidation pathways, an effect that is abolished when PGC-1α is knocked down. Physiologically, fasting and glucagon signaling increase hepatic KMT5C levels without altering RNF34, which contributes to elevated PGC-1α protein levels during nutrient-deprived states. In diabetic mice and humans, hepatic KMT5C expression is markedly higher, and liver-specific KMT5C depletion lowers fasting glucose by enhancing PGC-1α ubiquitin-mediated degradation, highlighting hepatic KMT5C as a potential therapeutic target for diabetes [321].

Bombassaro et al. [270] reported that A20, a deubiquitinase and E3 ligase, associates with PGC-1α and appears to protect it from degradation. In white adipose tissue from human obese subjects, PGC-1α and A20 levels were significantly lower than in lean individuals, and the amount of ubiquitinated PGC-1α was increased. Similar patterns were observed in different adipose depots of lean and obese mice. Inhibition of A20 in mice reduced PGC-1α and PPARγ levels in subcutaneous fat, leading to increased fasting glucose levels and impaired glucose tolerance. The findings in both humans and animal models suggest that A20 may act as a protective factor against PGC-1α ubiquitin-mediated degradation [270].

### SUMOylation

SUMO (small ubiquitin-like modifier) proteins are ubiquitin-like molecules that can be attached covalently to lysine residues, influencing various aspects of protein function [322]. This modification can alter subcellular and subnuclear localization, create new interaction surfaces for protein-protein interactions, and regulate transcriptional activity [322, 323]. In some cases, SUMOylation protects proteins from degradation when they share lysine residues that are also targeted by ubiquitination [323]. Like ubiquitination, the process is enzymatically regulated by activating (E1), conjugating (E2), and ligating (E3) enzymes, with SUMO-specific proteases (SENPs) reversing the modification [322, 323].

SUMOylation of PGC-1α is promoted by SUMO E3 ligases PIAS (protein inhibitor of activated STAT), primarily PIAS3 and PIAS1 [271]. Two lysine residues in PGC-1α, K183 and K645, match the SUMOylation consensus sequence (ψKXE, where ψ represents a residue containing a bulky hydrophobic side chain and X is any amino acid), but K183 is the main SUMO acceptor [271, 324]. Mutations at K183 (K183R) or in the SUMOylation consensus sequence (E185A) disrupt SUMOylation of PGC-1α to comparable levels. Relative to the wild-type protein, these mutant forms do not exhibit altered half-life, indicating that SUMOylation does not influence PGC-1α degradation [271]. Instead, K183R and E185A mutants demonstrate enhanced coactivator activity, suggesting that SUMOylation at K183 represses PGC-1α function. Moreover, expression of the nuclear corepressor RIP140 alters the nuclear distribution of wild-type PGC-1α, whereas the E185A mutant displays a more dispersed localization [271]. This suggests that disrupting SUMOylation reduces the sensitivity of PGC-1α to RIP140-mediated repression. Consequently, SUMOylation may serve as a backup mechanism for suppressing PGC-1α activity when acetylation-based inhibition is insufficient, since K183 is also targeted for acetylation by GCN5 [271].

Further insights into SUMOylation regulation come from studies on SUMO-specific proteases. Among the six SENPs in mammalian cells, SENP1 functions as a key de-SUMOylation enzyme for PGC-1α [272]. Increased SENP1 expression enhances the transcriptional coactivation activity of wild-type PGC-1α, but no additional activation occurs when SENP1 is coexpressed with the SUMOylation-deficient K183R PGC-1α mutant, suggesting that this mutation maximizes PGC-1α activity, rendering de-SUMOylation inefficient. Knocking down SENP1 reduces mRNA levels of transcripts encoding mitochondrial genes, including cytochrome c, ATP synthase subunit c, and UCP2, as well as transcription factors NRF-1/2β and ERRα. Ectopic SENP1 expression restores the levels of these transcripts to near wild-type levels. Additionally, SENP1 -/- cells exhibit fewer mitochondria, a lower mitochondrial DNA/nuclear DNA ratio, and decreased basal mitochondrial oxygen consumption. These findings highlight SENP1 as a crucial regulator of mitochondrial gene expression via PGC-1α de-SUMOylation [272].

### Integrative perspective of post-translational regulation of PGC-1α

**Fig. 6:**
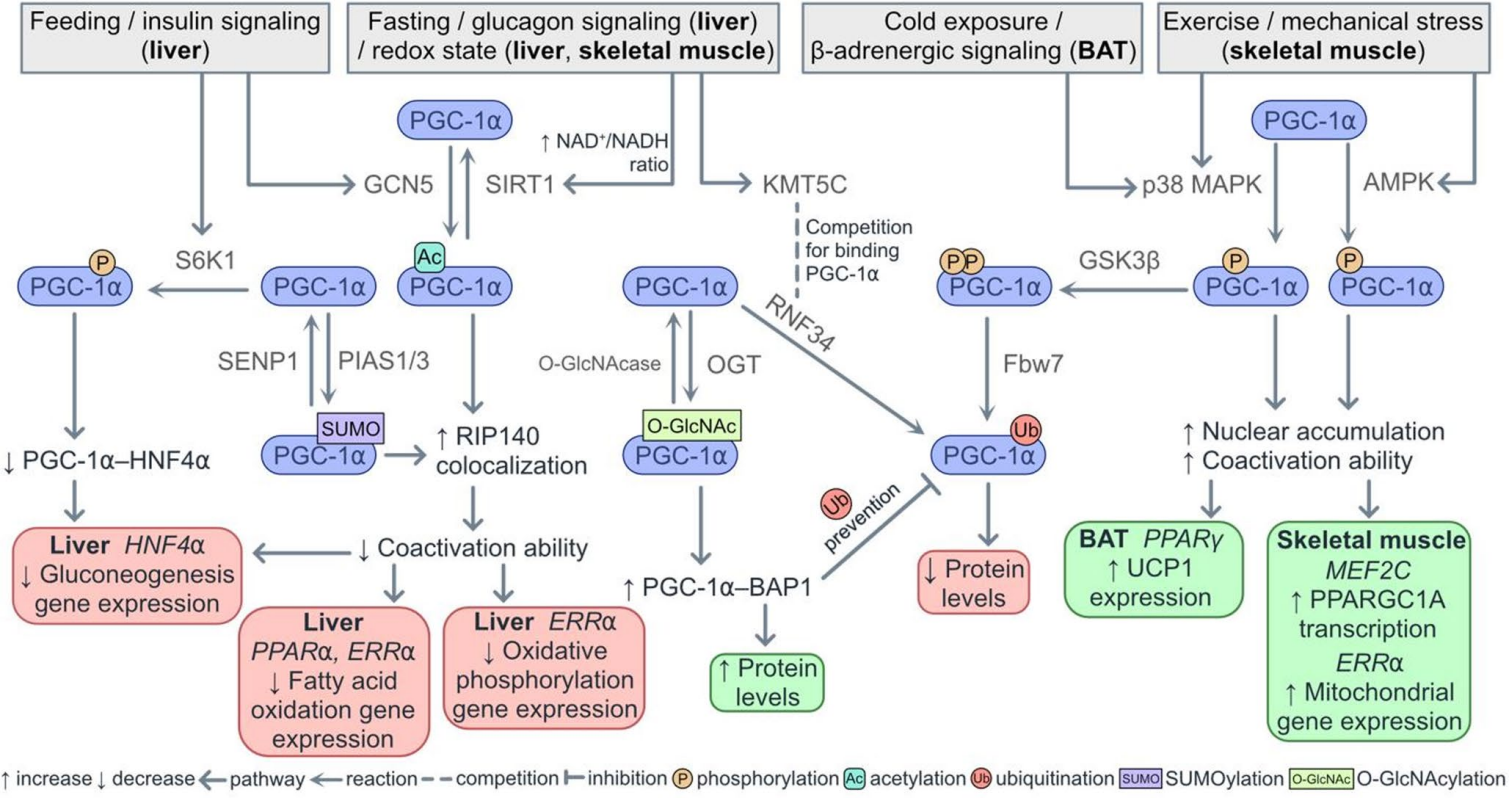
Core post-translational modifications that shape PGC-1α function by affecting activity, stability, localization, and/or interaction networks. Specific stimuli that trigger certain modifications are displayed at the top alongside a non-exhaustive list of tissues where they can occur (bold text). Outputs are shown at the bottom with boxes color-coded according to a positive (green) or negative (red) outcome on PGC-1α activity, together with key transcription factors (italics) whose transcriptional outputs are influenced by PGC-1α coactivation ability. Abbreviations: AMPK (AMP-activated protein kinase), BAP1 (BRCA1-associated protein 1), BAT (brown adipose tissue), ERRα (estrogen related receptor α), Fbw7 (F-box/WD repeat-containing protein 7), GCN5 (general control non-depressible 5), GSK3β (glycogen synthase kinase 3β), HNF4α (hepatocyte nuclear factor 4 α), KMT5 (lysine methyltransferase 5), MEF2C (myocyte-specific enhancer factor 2 C), OGT (O-linked N-acetylglucosaminyltransferase), p38 MAPK (p38 mitogen-activated protein kinase), PIAS1/3 (protein inhibitor of activated STAT 1/3), PPAR (peroxisome proliferator activated receptor), RIP140 (receptor interacting protein 140 kDa), RNF34 (ring finger protein 34), S6K1 (S6 kinase 1), SENP1 (SUMO-specific protease 1), SIRT1 (sirtuin 1), UCP1 (uncoupling protein 1)

Collectively, the post‑translational modifications described above reveal PGC‑1α as a signal‑responsive regulatory hub whose activity is shaped by coordinated, and often interdependent, modification events rather than by any single modification alone. Across tissues and metabolic contexts, post‑translational modifications converge on a limited set of functional outcomes: regulation of protein stability and turnover, selection of transcriptional partners, modulation of chromatin occupancy, and control of subcellular and subnuclear localization. This network of chemical modifications—summarized in Fig. 6 and Table 2—ultimately determines the magnitude, duration, and specificity of PGC‑1α–dependent transcriptional programs.

**Table 2:** Summary of PGC-1α post-translational modifications crosstalk categorized according to interaction type

Interacting modifications		Interaction type	Mechanistic basis	Functional outcomes
#1	#2			
Phosphorylation (AMPK)	Deacetylation (SIRT1)	Synergistic	AMPK-mediated phosphorylation increases SIRT1-mediated PGC-1α deacetylation	↑ PGC-1α coactivation ability
Phosphorylation (p38 MAPK)	Phosphorylation (GSK3β)	Sequential	Initial phosphorylation creates a recognition motif for subsequent GSK3β phosphorylation	↓ PGC-1α protein levels
Phosphorylation (GSK3β)	Ubiquitination (Fbw7)	Sequential	Phosphorylation creates degron motifs recognized by Fbw7 E3 ligase	
Acetylation (GCN5)	Deacetylation (SIRT1, HDAC3, HDAC1)	Antagonistic	Competing enzymatic modifications on lysine residues	↓ vs. ↑ PGC-1α coactivation ability
O-GlcNAcylation (OGT)	De-O-GlcNAcylation (O-GlcNAcase)	Antagonistic	Competing enzymatic modifications on Ser333	↓ vs. ↑ PGC-1α ubiquitination
O-GlcNAcylation (OGT)	Deubiquitination (BAP1)	Sequential	O-GlcNAcylation leads to increased PGC-1α–BAP1 association	↑ PGC-1α protein levels
SUMOylation (PIAS1, PIAS3)	De-SUMOylation (SENP1)	Antagonistic	Competing enzymatic modifications on Lys183	↓ vs. ↑ PGC-1α coactivation ability
SUMOylation (PIAS1, PIAS3)	Acetylation (GCN5)	Reinforcing	Competing enzymatic modifications on Lys183, yielding the same functional outcome (“backup mechanism”)	↓ PGC-1α coactivation ability (via increased RIP140 colocalization)
Ubiquitination (RNF34, Cullin1, Fbw7)	Deubiquitination (BAP1, A20)	Antagonistic	Competing enzymatic modifications on unidentified lysine residues	↓ vs. ↑ PGC-1α protein levels
Methylation (SET 7/9)	Demethylation (LSD1)	Antagonistic	Competing enzymatic modifications on Lys779	↑ vs. ↓ expression of genes under PGC-1α control

Several post‑translational modifications directly regulate PGC‑1α protein stability, allowing rapid adjustment of coactivator levels in response to environmental stimuli. Phosphorylation by p38 MAPK, O‑GlcNAcylation at Ser333, and deubiquitination by enzymes such as BAP1 act to stabilize PGC‑1α, whereas ubiquitination mediated by E3 ligases including Fbw7 and RNF34 promotes its degradation. Importantly, activation and degradation appear temporally coupled: AMPK or p38 MAPK-dependent phosphorylation primes PGC‑1α for SIRT1‑mediated deacetylation, simultaneously facilitating GSK3β-mediated phosphorylation (which requires a pre-phosphorylated residue for phosphorylation of adjacent threonine sites). GSK3β-dependent phosphorylation serves as a signal for intranuclear PGC-1α ubiquitination and subsequent degradation via the ubiquitin-proteasome pathway. These three post-translational modifications define a regulatory sequence in which activation, nuclear accumulation, and degradation are linked, ensuring that PGC-1α transcriptional activation of target genes remains transient and tightly controlled.

Post‑translational modifications also influence transcriptional specificity by modulating PGC‑1α interactions with selected transcription factors and coregulators. Acetylation by GCN5 represses PGC‑1α activity by redistributing it to inactive nuclear domains and weakening binding to factors such as HNF4α, whereas deacetylation by SIRT1 or HDACs restores coactivator ability in a tissue‑specific manner. Similarly, N‑terminal acetylation disrupts PGC‑1α interaction with PPARγ, while SUMOylation at Lys183 promotes recruitment of corepressors such as RIP140. The fact that Lys183 serves as a site of both acetylation and SUMOylation supports a competitive regulatory mechanism that enforces transcriptional repression under defined conditions. Insulin‑responsive kinases such as Akt2, S6K1, and Clk2 also impose selective repression by impairing chromatin binding without globally inhibiting the coactivation ability of the protein, allowing fine‑tuned, gene‑specific regulation. O‑GlcNAcylation further integrates nutrient availability into this network by modulating both PGC‑1α stability and its cooperation with transcriptional partners such as FOXO1.

Despite extensive characterization, several aspects of PGC‑1α post‑translational modification regulation remain unresolved, especially in the case of ubiquitination. While several E3 ligases have been implicated in PGC‑1α ubiquitin-mediated degradation, the specific Lys residues that are targeted by these ligases have not been determined, in contrast to other post-translational modifications, which have been characterized in greater detail. Additionally, conflicting models exist regarding the role of Fbw7 in PGC‑1α degradation, with studies differing on the dominant isoform, subcellular compartment, and magnitude of its contribution toward ubiquitin-mediated PGC-1α turnover. These discrepancies likely reflect methodological differences, including the use of tagged constructs, and cell‑type‑specific regulatory environments, as well as additional indirect effects mediated by pathways such as chaperone‑mediated autophagy. Rather than undermining the significance of ubiquitin‑mediated control, these findings highlight the context‑dependent nature of PGC‑1α degradation and the need for further studies to shed light on regulatory hierarchies involved in PGC‑1α turnover. Recent studies indicating that PGC‑1α turnover may be redirected between proteasomal and lysosomal pathways in response to environmental stress reinforce the view that degradation is dynamically coupled to metabolic context.

A related unresolved issue concerns the mechanisms governing PGC‑1α subcellular distribution. Although PGC‑1α is often described as a predominantly nuclear protein, growing evidence indicates that the nuclear‑to‑cytoplasmic ratio of the protein varies substantially across cell types and physiological conditions. Mechanical stimuli such as exercise, as well as metabolic or oxidative stress, have been associated with nuclear accumulation of PGC‑1α, concomitant with changes in its post‑translational modification state, including phosphorylation and deacetylation. Correlations between increased nuclear PGC‑1α content and activation of signaling pathways (p38 MAPK, AMPK, SIRT1) have been reported; however, these observations do not establish how PGC‑1α is actively transported, retained, or excluded from the nucleus at a molecular level.

At present, there is no direct evidence linking specific post‑translational modifications to defined nuclear import or export mechanisms for full‑length PGC‑1α. Progress in this area will likely require experimental designs that can systematically uncouple the various factors affecting PGC‑1α trafficking, for example through targeted mutagenesis or selective deletion of individual nuclear localization sequences, combined with analysis across cell types that display distinct basal localization patterns. Comparisons between full‑length PGC‑1α and truncated isoforms, integrated with perturbations of candidate import or retention pathways, could help distinguish direct effects on nucleocytoplasmic trafficking from secondary consequences of transcriptional activation. In parallel, bioinformatic analyses identifying sequence features shared with proteins subject to regulated cytoplasmic sequestration may aid in prioritizing candidate interaction motifs for experimental testing. Such approaches would enable a mechanistic framework for understanding how signaling‑dependent modifications are translated into context‑specific PGC‑1α localization and function.

Overall, PGC‑1α post‑translational modifications form a dynamic regulatory network that enables rapid adaptation to metabolic stress while preventing maladaptive or sustained activation. Understanding how these modifications cooperate, compete, or dominate under specific physiological and pathological states remains essential for interpreting PGC‑1α function and for guiding therapeutic strategies aimed at modulating its activity.

## Pharmacological modulation

Altered PGC-1α expression has been implicated in the development of numerous pathologies [57], including metabolic disorders [48], heart disease [43, 325], neurological disorders [32, 34, 35], and cancer [39, 40]. For example, in the muscle of type 2 diabetes mellitus (T2DM) subjects, oxidative phosphorylation genes are downregulated [326, 327], correlating with reduced levels of PGC-1α mRNA, which are lower by $\:\approx\:$ 20% relative to individuals with normal glucose tolerance [326]. In addition to altered expression, multiple PPARGC1A genetic variants have been associated with disease susceptibility. The common missense polymorphism Gly482Ser (rs8192678) is linked to increased risk of T2DM across multiple populations, including Asian, African, and European cohorts [328–330]. The Gly482Ser allele has also been associated with nonalcoholic fatty liver disease (NAFLD) [331] and hypertrophic cardiomyopathy [332]. In vitro studies indicate that this variant of the protein displays reduced stability, resulting in lower transcriptional coactivator activity on target genes encoding antioxidant proteins [333, 334]. The intronic variant rs2290602 has similarly been associated with NAFLD and reduced hepatic PGC-1α expression [335]. PPARGC1A polymorphisms have also been analyzed in the context of neurodegenerative diseases—the SNP rs7665116 correlates with age of onset in Huntington’s disease [336], rs11737023 has been linked with age of death in amyotrophic lateral sclerosis in a sex-dependent manner [337], and several rare pathogenic PPARGC1A variants have been connected to early-onset or familial Parkinson’s disease [338]. Increased susceptibility to various cancer types for carriers of specific PPARGC1A SNPs has also been reported, in particular for familial breast cancer (rs3736265) [339], colorectal cancer (rs3774921) [340], and esophageal squamous cell carcinoma (rs3736265) [341].

Given its central role in metabolic regulation, PGC-1α represents an attractive therapeutic target for conditions characterized by metabolic dysregulation, mitochondrial dysfunction, or excessive ROS production, such as T2DM, obesity [342], Alzheimer’s disease [343], Huntington’s disease [344], and Parkinson’s disease [345]. Human genetic studies linking PPARGC1A variants to metabolic and neurodegenerative disease further support the therapeutic relevance of modulating PGC‑1α activity, while highlighting the need for precise and context‑dependent pharmacological strategies (i.e., personalized medicine). However, the identification of direct PGC‑1α‑modulating small molecules has proven challenging due to its intrinsically disordered nature, which implies a lack of traditional ligand-binding pockets typically exploited by structure-guided drug design approaches. This challenge is compounded by the limited availability of experimentally derived structural information: with the exception of leucine-rich motifs crystallized in complex with nuclear receptors, no high-resolution structural data have been reported for PGC‑1α. Consequently, most efforts to identify PGC‑1α modulators have relied on phenotypic or reporter‑based high‑throughput screening (HTS) strategies rather than rational design [346]. Additionally, none of the compounds reported so far have a confirmed mechanism of action that involves engaging directly with the protein through defined structural interfaces. Instead, most act in an indirect manner, influencing PGC‑1α expression or specific post-translational modifications.

This section describes key synthetic and natural small molecules (Table 3) identified through targeted screening approaches designed to specifically alter PGC-1α activity, or that demonstrate a measurable biological effect dependent on the presence of PGC‑1α protein or intact PPARGC1A gene in in vitro or in vivo models. Metabolic modulators with widespread effects or pleiotropic compounds not discovered through PGC‑1α‑directed screening efforts—including natural polyphenolic compounds (e.g., resveratrol), hormones (melatonin), neuropeptides (cerebrolysin, Semax), are comprehensively covered in other reviews [57, 347, 348].

For clarity, compounds listed in Table 3 are grouped according to the mode of functional modulation of PGC‑1α as: (i) indirect activators that increase PPARGC1A transcription through upstream signaling pathways; (ii) compounds that alter PGC-1α transcriptional coactivation ability by inducing specific post-translational modifications; (iii) putative stabilizers that increase PGC‑1α protein levels without a defined molecular target. All small molecules remain at the preclinical stage at the time of writing, and for most of them the precise molecular mechanisms of PGC‑1α modulation have yet to be fully elucidated.

**Table 3:** Summary of PGC-1α small molecule modulators classified by mode of functional modulation and originally targeted tissue. All listed compounds are, at the time of writing, in the preclinical development stage

Molecule	Origin	Development Stage	Mode of modulation	Targeted tissues/cells	Refs.
ZLN005	Synthetic	Preclinical, Lead Discovery	Indirect activation via increased PGC-1α transcript levels	Skeletal muscle cells	[349]
SR18292	Synthetic	Preclinical, Lead Discovery	Indirect inhibition by inducing PGC-1α acetylation	Hepatocytes	[350–352]
AM31, AM73, AM79, AM80, AM89	Synthetic	Preclinical, Hit Validation	Putative stabilization, increasing PGC-1α protein levels	Brown adipocytes	[353]
Atractylenolide III, Atractylenolide I	Natural	Preclinical, Pharmacological Characterization	Indirect activation by inducing PGC-1α deacetylation	Dopaminergic neurons and intestinal epithelial cells	[354, 355]
Matrine	Natural	Preclinical, Pharmacological Characterization	Indirect activation via increased PGC-1α transcript levels	White adipocytes	[356]
HN-001 (4,5-dimethoxycandidusin A)	Natural	Preclinical, Early Discovery	Indirect activation via increased PGC-1α transcript levels	White adipocytes	[357]

### ZLN005 – indirect PGC-1α activator

**Fig. 7:**
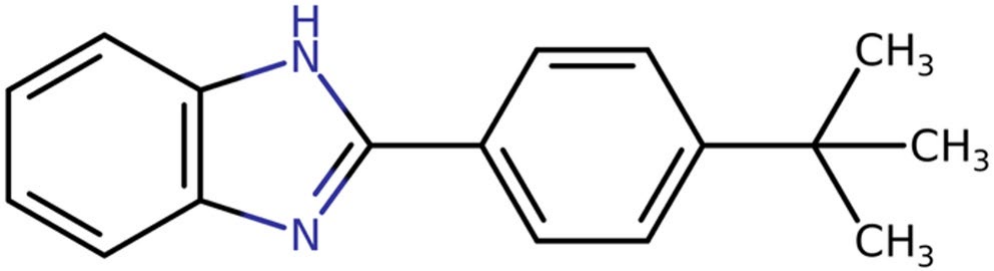
Molecular structure of ZLN005, a compound capable of upregulating PGC-1α, discovered by Zhang et al. [358]

Zhang et al. [358] identified the compound ZLN005 as an indirect activator of PGC-1α capable of increasing PGC-1α expression in skeletal muscle cells. Using an automated high-throughput screening assay, 48,000 compounds were tested with regards to their ability to induce PGC-1α expression. Primary analysis revealed that the compound ZLN027 was able to upregulate PGC-1α but was cytotoxic. A structurally similar, non-toxic compound—ZLN005 (Fig. 7)—was identified and shown to increase PGC-1α mRNA levels by 3-fold, along with upregulation of GLUT4, ERRα, cytochrome c, and acetyl-CoA oxidase in myotubes [358].

ZLN005 is proposed to act by weakly uncoupling mitochondrial respiration, which increases the cellular AMP/ATP ratio and activates AMPK. This leads to the phosphorylation of PGC-1α at T177 and S538. Phosphorylated PGC-1α then binds to MEF2C to increase its own gene expression in a positive feedback loop [358]. In an animal model of type 2 diabetes (db/db mice), treatment with ZLN005 lowered fasting blood glucose, improved insulin sensitivity, and decreased plasma triglycerides, cholesterol, and non-esterified fatty acids, without affecting body weight or food intake. Despite acting as a PGC-1α activator in skeletal muscle cells, in the liver of treated db/db mice, PGC-1α and gluconeogenic genes were downregulated, while the expression of mitochondrial oxidative phosphorylation genes remained unaffected [358].

Beyond diabetes, ZLN005 has been studied for a variety of conditions, demonstrating protective effects against glucose-induced cardiomyocyte cytotoxicity [357], neuroinflammation in perioperative neurocognitive disorders [359], ischemia-reperfusion injuries [360, 361], neurotoxin-induced mitochondrial dysfunction [362], drug-induced cardiomyopathy [363], ischemia-induced neuronal injury [364] and traumatic brain injury [365]. It has also shown potential in chronic kidney disease by mitigating mitochondrial dysfunction [366].

In addition to its potential as a pharmaceutical agent, ZLN005 has been used to promote the maturation of human pluripotent stem-cell derived cardiomyocytes (hPSC-CMs) [367]. These cells typically resemble mid-gestation fetal cardiomyocytes, and this underdeveloped phenotype limits their usefulness in regenerative medicine and cell replacement therapies [368]. ZLN005 treatment for 48 h increased PGC-1α mRNA and protein levels, upregulating key genes involved in mitochondrial biogenesis, oxidative phosphorylation, and fatty acid oxidation. Moreover, structural genes were upregulated, leading to the acquisition of a more mature morphology, with improved electrical activity and enhanced Ca^2+^ kinetics [367].

Although ZLN005 has shown therapeutic efficacy across multiple pathological contexts in vitro and in vivo, its safety profile and therapeutic window remain insufficiently characterized. Notably, a recent study reported that sustained ZLN005 administration (14 days) following myocardial infarction exacerbated cardiac dysfunction in an animal model of the disease [369]. More specifically, ZLN005 treatment increased cardiomyocyte death under ischemic conditions and was associated with reduced intracellular ATP levels and decreased mitochondrial content in vitro [369]. These findings raise particular concern given prior evidence that supraphysiological PGC‑1α expression can be deleterious in specific tissues. For example, forced cardiac‑specific overexpression of PGC‑1α in mice results in dilated cardiomyopathy, attributed to excessive mitochondrial biogenesis and displacement of myofibrils [26]. Inducible overexpression in the hearts of adult mice causes a more modest increase in the number of mitochondria and less myofibrillar degeneration, but ultrastructural mitochondrial abnormalities are observed, accompanied by the development of cardiomyopathy, which is largely reversible upon cessation of overexpression [370].

Similarly, high‑level PGC‑1α overexpression in skeletal muscle (≥ 10‑fold increase in mRNA) increases mitochondrial abundance and uncoupled respiration but markedly reduces intracellular ATP levels, leading to muscle atrophy, particularly in type II fiber‑rich muscles [371]. Taken together, these studies suggest that sustained or excessive PGC‑1α activation may impose tissue‑specific risks that warrant careful consideration in the translational development of ZLN005.

### SR18292 – indirect PGC-1α inhibitor

**Fig. 8:**
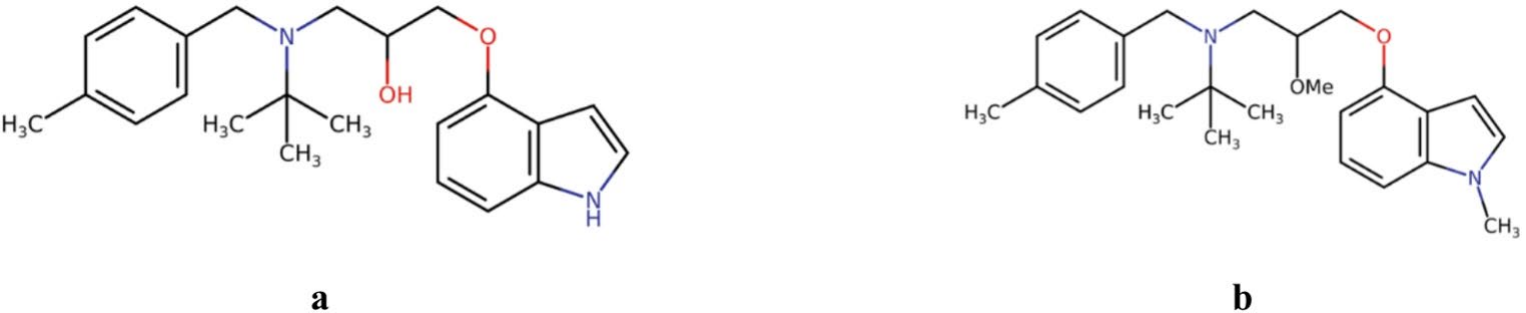
Molecular structure of PGC-1α inhibitors: **a** SR18292 and **b** its analog SR20259 lacking β-adrenergic antagonism [349–351]

Sharabi et al. [349] developed a high-throughput screening platform to identify small molecules that enhance PGC-1α acetylation, aiming to discover new drug candidates for T2DM treatment. Screening 350,000 compounds, U-2 OS cells (osteosarcoma cell line) coexpressing PGC-1α and GCN5 were used to assess acetylation changes. Compounds that increased lysine acetylation by over 50% were further tested for their ability to suppress gluconeogenic genes PCK1 and G6PC in hepatocytes. The most potent compounds were assessed in a final assay which measured reduction in glucose release in hepatocytes ectopically expressing PGC-1α. Due to limited commercial availability, an analog of one of the most potent compounds was synthesized and found to be equally effective, SR18292 (Fig. 8a) [349].

SR18292 was tested in an animal model of diabetes, high fat diet (HFD)-fed mice. The treated mice exhibited significantly lower levels of fasting blood glucose, enhanced glucose tolerance and insulin response, without changes in food intake, body weight, and insulin levels. Additionally, no liver toxicity was observed after 14 days of treatment. The compound selectively inhibited gluconeogenesis without altering mitochondrial gene expression [349].

Lin et al. [350] conducted structure-activity relationship studies on SR18292, modifying its chemical scaffold to identify functional groups essential for its biological activity. It was also shown that SR18292 exhibits weak β-adrenergic antagonism, mildly suppressing lipolysis in adipocytes—albeit far less potently than classical β-blockers, e.g. propranolol. To refine the therapeutic profile of SR18292, over 20 analogs were synthesized and tested. Elimination of β-adrenergic activity was accomplished via O-methylation of the secondary alcohol. This modification also enhanced suppression of glucagon-induced gluconeogenesis compared to the parent compound; however, when accompanied by N-methylation of the pyrrole ring (Fig. 8b), this suppressive effect was comparable to SR18292 [350].

The exact molecular target of SR18292 remains unclear. While it increases PGC-1α acetylation, this effect is independent of SIRT1 and does not interfere with GCN5 catalytic activity. The inhibition of gluconeogenesis is achieved by a reduction in the ability of PGC-1α to coactivate HNF4α. This was evidenced by decreased promoter occupancy of the gluconeogenic genes PCK1 and G6PC by HNF4α in treated cells [349]. It is hypothesized that SR18292 may enhance PGC-1α–GCN5 interaction by targeting an upstream regulator that modifies GCN5 through post-translational modifications and consequently affects binding to PGC-1α [349].

Mutlu et al. [351] recently demonstrated that SR18292 enhances the oxidation of gluconeogenic substrates, such as lactate and glutamine, through the tricarboxylic acid (TCA) cycle in a PGC-1α-dependent manner. By promoting substrate oxidation, SR18292 prevents their accumulation, which could otherwise fuel *de novo* lipogenesis and contribute to hepatic steatosis. Additionally, SR18292 increases glucose oxidation by enhancing the acetylation of PCK1. Normally, PCK1 favors the conversion of oxaloacetate (OAA) to phosphoenolpyruvate (PEP). However, under high glucose conditions, acetylation shifts PCK1 activity toward the reverse reaction, converting PEP back to OAA. This process supplies OAA to the TCA cycle, driving lactate oxidation. Thus, SR18292 suppresses hepatic glucose production by inhibiting PGC-1α-dependent gluconeogenic gene expression while preserving fatty acid oxidation and mitochondrial function. At the same time, it redirects gluconeogenic precursors into oxidative pathways rather than lipid biosynthesis, preventing hepatic lipid accumulation [351].

Beyond its anti-diabetic effects, SR18292 has demonstrated anti-cancer properties. In multiple myeloma cells, SR18292-mediated inhibition of PGC-1α resulted in downregulated oxidative phosphorylation genes, impairing cancer cell proliferation due to energy depletion [372]. In cervical squamous cell carcinoma, SR18292 synergized with the natural compound cyanidin-3-O-glucoside, amplifying ROS accumulation and promoting apoptosis [373].

Overall, while SR18292 demonstrates robust metabolic efficacy in preclinical models, several factors currently limit assessment of its translational potential. These include the absence of a precisely defined molecular target underlying its effects on PGC‑1α acetylation, the presence of secondary activities such as weak β‑adrenergic antagonism, and the limited availability of detailed tissue‑specific toxicity and long‑term safety data. In addition, because SR18292 modulates acetylation‑dependent processes, the possibility of effects on pathways beyond PGC‑1α—such as other transcriptional coactivators or metabolic regulators—cannot yet be excluded. Addressing these issues will be important for evaluating specificity, safety, and suitability for chronic therapeutic use.

### AM31, AM73, AM79, AM80, AM89 – PGC-1α stabilizers

**Fig. 9:**
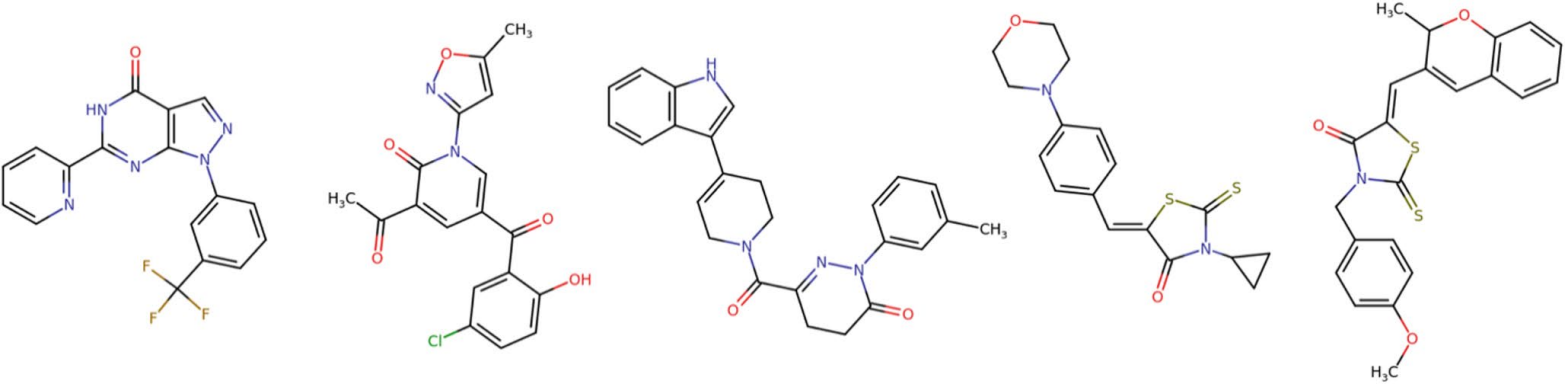
PGC-1α stabilizers discovered by Petterson-Klein et al. [352]. From left to right: AM31, AM73, AM79, AM80, AM89

Petterson-Klein et al. [352] developed a high-throughput screening assay to identify PGC-1α stabilizers, based on the literature evidence that PGC-1α is an intrinsically disordered protein [90]. A total of 7040 small molecule compounds were tested in the primary assay, using HEK293 cells ectopically expressing PGC-1α. Compounds that were able to maintain the highest PGC-1α levels were further assessed in a secondary assay using mouse brown adipocyte cell line. Then, the ability of the compounds to stabilize endogenous PGC-1α as well as induce the expression of known PGC-1α target genes was measured. Seventeen compounds were able to induce UCP1 expression and PGC-1α accumulation, and of these, five increased UCP1 5-fold over control: AM31, AM73, AM79, AM80, and AM89 (Fig. 9) [352].

In brown adipocytes, an increase in basal mitochondrial respiration was observed with all compounds except AM89. Compound AM80 was the only to show a small increase in maximal respiration after treatment with FCCP (a mitochondrial oxidative phosphorylation uncoupler), indicating the presence of more mitochondria or mitochondrial protein. The exact mechanism of action of these PGC-1α stabilizers was not determined. It is hypothesized that the compounds may interact with the protein itself, stabilizing it in a conformation that enhances its stability and makes it less susceptible to degradation [352]. However, direct binding to PGC‑1α has not been demonstrated, and stabilization may instead reflect indirect effects on protein turnover pathways.

### Natural compounds capable of modulating PGC-1α activity

#### Atractylenolides (I and III)

**Fig. 10:**
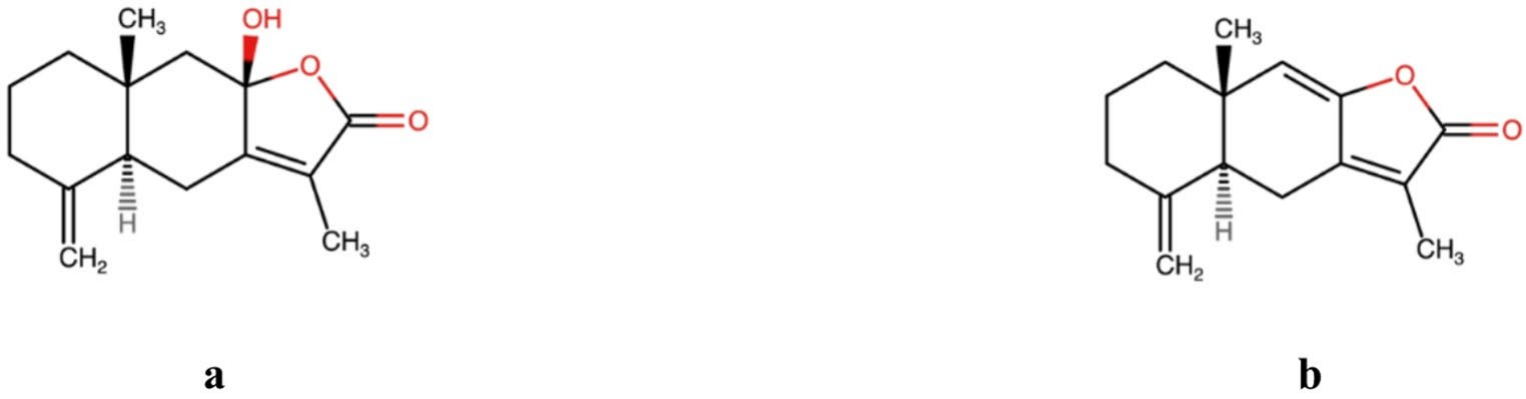
Natural PGC-1α modulators extracted from the roots of *Atractylodes macrocephala*: **a** AT III [353] and **b** AT I [354]

Han et al. [353] demonstrated that atractylenolide (AT) III (Fig. 10a), a bioactive compound extracted from the roots of *Atractylodes macrocephala*—a traditional Chinese medicinal herb—can alleviate mitochondrial dysfunction in ulcerative colitis (UC). AT III achieves this by promoting PGC-1α deacetylation in intestinal epithelial cells through the AMPK/SIRT1 pathway. Using a mouse model of UC, AT III treatment increased mitochondrial DNA copy number and enhanced the activities of electron transport chain complex I and complex IV in the colons of UC mice. Additionally, AT III restored the expression levels of mitochondrial-related proteins (PGC-1α, NRF-1/2, and TFAM) and reversed the reduction of AMPK and SIRT1 levels in the colons of UC mice [353]. AT I (Fig. 10b), a compound extracted from the same herb, was found to have therapeutic effects in a mouse model of Parkinson’s disease, by mitigating oxidative stress and increasing survivability of dopaminergic neurons [354]. These neuroprotective effects were blunted by administrating a SIRT1 inhibitor (EX527), which points to a mechanism of action of AT I that requires SIRT1-mediated PGC-1α deacetylation for downstream activation of oxidative stress response [354]. Given the structural similarity between AT III and AT I and the comparable treatment outcomes (increased PGC-1α deacetylation), it is likely AT I/III target SIRT1 directly or another biomolecule upstream of SIRT1.

Pharmacokinetic studies in rodents indicate that atractylenolides exhibit favorable absorption profiles, characterized by rapid intestinal uptake and relatively slow elimination following oral administration [374]. Absorption appears to occur predominantly Style change has been done. Please check.via passive diffusion in the intestine, with AT III achieving higher mean plasma concentrations compared to AT I and AT II under similar dosing conditions [375]. Following systemic absorption, atractylenolides display compound‑specific tissue distribution patterns: AT I preferentially accumulates in the liver, kidney, spleen, and cerebellum, whereas AT III shows higher distribution to the lung, cerebellum, and heart [374, 376]. These properties support systemic bioavailability but also suggest tissue‑selective exposure that may be relevant for both efficacy and safety.

Despite promising anti‑inflammatory and antioxidant effects in preclinical models, important translational gaps remain. Most of the available evidence for atractylenolides derives from cell‑based assays and animal studies, with a marked lack of systematic toxicity and safety characterization in humans [377]. Emerging preclinical safety data further indicate potential compound‑specific risks: in zebrafish embryo models, exposure to AT I induced developmental abnormalities and hepatotoxicity, whereas AT III did not elicit detectable toxic phenotypes [378]. Hepatic injury associated with AT I exposure correlated with downregulation of hepatic drug‑metabolizing enzymes, suggesting impaired detoxification capacity [378]. Together, these findings indicate that while atractylenolides—particularly AT III—hold pharmacological promise, careful compound‑specific safety evaluation will be essential before clinical translation can be considered.

#### Matrine

**Fig. 11:**
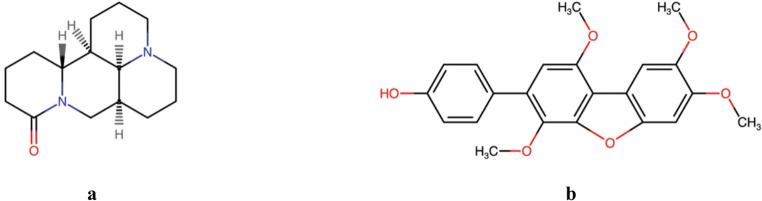
Natural PGC-1α modulators discovered through high-throughput screening: **a** matrine [355] and **b** HN-001 (4,5-dimethoxycandidusin A) [356]

Li et al. [355] screened 428 natural compounds to identify potential activators of the heat shock factor 1 (HSF1)/PGC-1α axis as therapeutic candidates for obesity and metabolic disorders. The most potent compound was matrine (Fig. 11a), an alkaloid extracted from *Sophora flavescens*, a traditional Chinese medicinal herb known for its hepatoprotective properties. Chromatin immunoprecipitation analysis revealed that matrine enhances HSF1 binding to the PGC-1α promoter, increasing its transcription. In mature adipocytes, matrine treatment increased UCP1 expression, enhanced mitochondrial oxidative capacity, and suppressed genes involved in fatty acid synthesis. This led to a reduction in cellular lipid accumulation, evidenced by smaller lipid droplets. These effects were entirely dependent on PGC-1α, as adipocytes lacking PGC-1α showed no changes in gene expression upon matrine treatment [355]. In vivo evidence supporting the metabolic efficacy of matrine has been provided in a high‑fat, high‑cholesterol diet–induced obesity mouse model. In this study, matrine administration (100 mg/kg for 6 weeks) improved glucose tolerance and insulin sensitivity and increased whole‑body heat production, without affecting daily food intake [355].

However, matrine is known to exert pleiotropic biological effects beyond the HSF1/PGC‑1α pathway. Extensive literature describes its antitumor activity, which is mediated through modulation of multiple signaling networks governing cell‑cycle arrest, apoptosis, autophagy, and cell migration [379]. While such multifunctionality may be advantageous in certain disease contexts, it complicates mechanistic attribution and presents challenges for defining a clear therapeutic window when matrine is considered a PGC‑1α–targeted metabolic modulator.

From a pharmacokinetic perspective, matrine exhibits less favorable properties compared with other natural PGC‑1α modulators discussed herein. Oral bioavailability in rats is relatively low (approximately 17–18% at 2 mg/kg), and systemic exposure is limited by a short elimination half‑life of ≈ 1.5 h [380]. Tissue distribution studies indicate preferential accumulation in the liver following oral administration [381], suggesting low bioavailability is likely a byproduct of extensive first‑pass metabolism. Overall, these characteristics may constrain clinically feasible dosing regimens. Additionally, prolonged matrine administration in rodents (90 days at ≈ 69 mg/kg) has been reported to induce mild histological changes in the liver (centrilobular hypertrophy) [382]. While this finding is common in chemical toxicity studies using rodents and can be interpreted as an adaptive hepatic response in the absence of other markers of liver injury [383], it nonetheless highlights potential dose- and duration-dependent liver liabilities. Other preclinical studies have reported neurotoxicity and reproductive toxicity associated with matrine use [384], indicating the need for thorough systemic safety characterization.

Considering these limitations, considerable effort has been devoted to improving matrine’s therapeutic efficacy. Strategies include the development of advanced drug‑delivery systems, such as liposomal formulations, aimed at enhancing bioavailability and reducing off‑target toxicity [385–387]. In parallel, chemical modification approaches have yielded matrine derivatives with improved pharmacological activity and safety profiles, which have been comprehensively reviewed elsewhere [388].

#### HN-001

Using the same screening system as Li et al. [355], Rao et al. [356] identified a *p*-terphenyl compound derived from the marine fungus *Aspergillus* c1. sp. as another activator of the HSF1/PGC-1α axis. This compound, referred to by the authors as HN-001 (Fig. 11b) [356], is also known as 4,5-dimethoxycandidusin A and is considered a likely derivative of candidusin A, another fungus-derived bioactive *p*-terphenyl [389]. In white adipocytes, treatment with HN-001 increased HSF1 occupancy at the PGC-1α promoter, leading to enhanced PGC-1α transcription and upregulation of genes involved in mitochondrial biogenesis (NRF-2), oxidative phosphorylation (COX4), fatty acid oxidation (CPT-1β), and thermogenesis (UCP1). The effects of HN-001 were dependent on HSF1, as HSF1 knockout adipocytes exhibited a significantly reduced metabolic response when treated with the compound. In diet-induced obese mice, eight weeks of HN-001 treatment resulted in reduced body weight, improved metabolic profiles, and increased thermogenesis, as confirmed by body temperature analysis and cold tolerance tests, indicating higher energy expenditure [356].

Despite promising preclinical findings, HN‑001 remains poorly characterized with respect to pharmacokinetics and safety. More generally, *p*-terphenyl compounds have been reported to display diverse biological activities, including cytotoxic, antimicrobial, and antioxidant effects [390]. While no toxicological studies have been reported specifically for HN‑001, structurally related candidusin compounds provide some contextual precedent: candidusin A and B have been shown to exert cytotoxic effects in sea urchin embryo models, with candidusin B inhibiting DNA and RNA synthesis [391]. In addition, several marine‑derived *p*-terphenyls have demonstrated antiproliferative activity in cancer cell lines [392]. Although such observations cannot be extrapolated directly to HN‑001, they emphasize the need for dedicated pharmacological and toxicological evaluation.

Overall, relative to other natural compounds discussed in this review, HN‑001 should be regarded as being at an earlier stage of preclinical development. Comprehensive characterization of its pharmacokinetic properties, tissue distribution, and safety profile will be necessary before its therapeutic potential as a modulator of the HSF1/PGC‑1α axis can be more meaningfully assessed.

## Conclusions

Since its discovery, PGC-1α has emerged as a central integrator of metabolic adaptation, coordinating transcriptional programs through a combination of structurally encoded interaction motifs, context-dependent partner selection, and extensive post-translational regulation. Rather than functioning as a simple transcriptional amplifier, PGC‑1α operates as a dynamic regulatory scaffold whose output is determined by the interplay between its modular domains, the tissue-specific availability of specific transcription factors, and signaling‑driven modifications that govern stability, localization, and temporal activity. As reviewed here, differences in binding topology (e.g., L2/LXXLL‑ versus L3‑mediated interactions), ligand‑dependent versus signal‑dependent recruitment, and canonical versus non‑canonical functions collectively explain how PGC‑1α can orchestrate distinct transcriptional outcomes across tissues and physiological states.

An important theme that emerges is that PGC‑1α regulation is inherently context‑restricted. Tissue‑specific expression patterns of transcription factor partners (such as ERRs, PPARs, MEF2s and HNF4α), differential engagement of post‑translational modification pathways, and variable subcellular distribution together constrain PGC‑1α activity such that global activation is neither uniform nor desirable. This framework helps reconcile seemingly contradictory observations in the literature, including the reported pro‑ and anti‑tumor roles of PGC‑1α, which reflect differences in partner usage, duration of activation, and metabolic state rather than intrinsic oncogenic or tumor‑suppressive properties of the coactivator itself.

Despite substantial progress, several fundamental gaps continue to limit both mechanistic understanding and translational advance. Among the most pressing is the lack of a clear molecular framework explaining how post‑translational modifications regulate PGC‑1α subcellular localization. Numerous studies report phosphorylation- or deacetylation-associated nuclear accumulation under specific stimuli, yet the mechanisms governing nuclear import, retention, or export of full‑length PGC‑1α remain unresolved. More broadly, structural information on PGC‑1α is significantly sparse: with the exception of low‑resolution biophysical analyses published over a decade ago and short peptide co‑crystal structures derived from nuclear receptor complexes, no detailed structural characterization of the protein has been achieved experimentally. This lack of information contrasts sharply with the central role of PGC‑1α across tissues and disease states and represents a major constraint on identifying potential sites amenable to small‑molecule binding.

Addressing these knowledge gaps will require renewed emphasis on fundamental biochemical and structural investigations. Systematic mapping of protein-protein interaction regions required for partner binding, nucleocytoplasmic trafficking, and regulated turnover—using targeted mutagenesis or high-resolution structural methods such as solution nuclear magnetic resonance (NMR) spectroscopy—remains an essential prerequisite for meaningful mechanistic interpretation. While disease-focused and intervention-driven studies have greatly expanded the range of observed PGC‑1α functions across different biological contexts, the underlying mechanisms governing these effects are still incompletely defined. As a result, interpretation of these findings is often based on correlations rather than direct mechanistic evidence. Prioritizing studies that first establish the molecular basis of PGC‑1α regulation, and subsequently examine their relevance in disease models, will be essential to build a more coherent and predictive understanding of its function and to support the rational development of therapeutic strategies.

## Data Availability

Not applicable.
